# Targets of the transcription factor Six1 identify previously unreported candidate deafness genes

**DOI:** 10.1242/dev.204533

**Published:** 2025-04-14

**Authors:** Ramya Ranganathan, Fereshteh Sari, Scarlet Xiaoyan Wang, Alexandre Thiery, Ailin Leticia Buzzi, Rosalinda Guerra, Sally A. Moody, Andrea Streit

**Affiliations:** ^1^ Centre for Craniofacial and Regenerative Biology, King's College London, London SE1 9RT, UK; ^2^ Department of Anatomy & Cell Biology, George Washington University School of Medicine and Health Sciences, Washington, DC 20052, USA

**Keywords:** Ear development, Otic placode, Branchio-oto-renal Syndrome, Deafness

## Abstract

Branchio-otic (BOS) and branchio-oto-renal (BOR) syndromes are autosomal dominant disorders featuring multiple birth defects including ear, renal and branchial malformations. Mutations in the homeodomain transcription factor SIX1 and its co-factor EYA1 have been identified in about 50% of individuals with BOS or BOR, while causative mutations are unknown in the other half. We hypothesise that SIX1 target genes represent new BOS and BOR candidates. Using published transcriptomic and epigenomic data from chick ear progenitors, we first identify putative Six1 targets. Next, we provide evidence that Six1 directly regulates some of these candidates: Six1 binds to their enhancers, and functional experiments in *Xenopus* and chick confirm that Six1 controls their expression. Finally, we show that most putative chick Six1 targets are also expressed in the human developing ear and are associated with known deafness loci. Together, our results not only characterise the molecular mechanisms that mediate Six1 function in the developing ear, but also provide new candidates for human congenital deafness.

## Teaser

**Highlighted Article:** The identification of previously unknown Six1 targets, the molecular mechanisms that mediate Six1 function in the developing ear and potential candidates for human congenital deafness.

## INTRODUCTION

The transcription factor *Six1* and its co-factor *Eya1* are crucial for the development of different organs, including the kidney, neural crest derivatives and sense organs in the vertebrate head (Brugmann et al., 2004; Chen et al., 2009; Christophorou et al., 2009; Ikeda et al., 2007; Laclef et al., 2003a; Ozaki et al., 2004; Xu et al., 1999, 2003; Zheng et al., 2003; Zou et al., 2008, 2004, 2006b). In humans, mutations in these genes cause branchio-otic (BO) or branchio-oto-renal (BOR) syndrome, an autosomal dominant disease associated with hearing loss, branchial fistula and kidney defects (Neal et al., 2024; Smith, 2018). In mice, deletion of either Six1 or Eya1 leads to phenotypes consistent with the human abnormalities resulting in arrested ear formation, lack of the metanephric kidney and other developmental defects (Chen et al., 2009; Ikeda et al., 2007; Laclef et al., 2003a; Ozaki et al., 2004; Xu et al., 1999, 2003, 2002; Zheng et al., 2003; Zou et al., 2008, 2004, 2006b). However, only about 50% of humans presenting with BO and/or BOR (BO/BOR) features carry mutations in the SIX1 or EYA1 coding or regulatory regions. Therefore, the genetic causes for the remaining half of cases are unknown (Kimberling et al., 2011; Klingbeil et al., 2017; Lindau et al., 2014; Morisada et al., 2014; Sanggaard et al., 2007; Unzaki et al., 2018). Downstream effectors of Six1 are good candidates to be associated with BO/BOR syndrome or non-syndromic forms of hearing loss. Such targets have been identified in nephrons and in developing hair cells (Li et al., 2020; O'Brien et al., 2018). However, Six1 and its co-factor Eya1 play a much earlier role in controlling growth and cell specification in the ear primordium, i.e. the otic vesicle (Ozaki et al., 2004; Xu et al., 1999; Zou et al., 2006b). We therefore aimed to identify previously unreported Six1 targets in inner ear progenitors.

Six1 acts as a transcriptional activator or repressor, depending on the availability of co-factors. Upon binding to Eya1, Six1 switches from repressor to activator through the recruitment of co-activators, while, together with factors like Sobp and Dach1, it mediates transcriptional repression (Brugmann et al., 2004; Li et al., 2003; Ohto et al., 1999; Silver et al., 2003; Tavares et al., 2021). We have previously characterised transcripts enriched in the inner ear progenitors as well as many regulatory regions that control their expression (Buzzi et al., 2022; Chen et al., 2017). Taking advantage of these data, we now identify putative Six1 target genes in the otic placode, i.e. the primordium of the entire inner ear. We selected four such targets for further investigation: *Rnf150*, *Zbtb16*, *Znf385c* and *Pick1*. We show that in both chick and *Xenopus*, these genes are co-expressed with, and regulated by, Six1*.* We provide evidence that Six1 binds to regulatory regions associated with these genes, and that Six1 motifs are required for normal enhancer activity *in vivo*.

Finally, we explored whether putative Six1 targets are also expressed in the human ear. Much of our knowledge of early ear development is based on studies from animal models. However, how this process is controlled in human embryos remains unclear. Anatomical approaches provide some insight into the morphological events of ear formation (de Bakker et al., 2016; Yasuda et al., 2007), while the transcriptional changes that accompany ear development are poorly characterised. In humans, the otic placode invaginates around day 20 (Carnegie stage CS11), followed by vesicle formation at CS13, followed by cochlear duct outgrowth from CS15 onwards (de Bakker et al., 2016; Doda et al., 2023; Roccio et al., 2018). While recent single cell transcriptomics have characterised cell diversity and gene expression at later stages, after the cochlea has begun to grow out (Roccio et al., 2018; van der Valk et al., 2023; Yu et al., 2019 preprint), molecular characterisation of otic vesicle stages is currently limited to a few markers detected by immunostaining (Doda et al., 2023). Here, we present the first transcriptome analysis of human embryonic otic vesicles from CS13 and CS14. We show that many of the putative chick Six1 targets are indeed expressed in human embryonic ears *in vivo*. Interestingly, the genomic location of many putative Six1 targets coincides with human deafness loci where the causative gene remains to be identified, introducing previously unreported candidate deafness genes for BOR or other forms of deafness.

## RESULTS

### Identification and expression of putative Six1 targets in inner ear progenitors

During development, the entire inner ear arises from a pool of progenitors located in the otic placode, which is a transient patch of ectoderm next to the hindbrain. The placode invaginates to form a vesicle, which is then transformed into the complex architecture of the inner ear (Barald and Kelley, 2004; Groves and Fekete, 2012; Wu and Kelley, 2012). To predict direct Six1 target genes at early developmental stages, we took advantage of our transcriptomic and epigenomic data, which identified 279 genes enriched in the otic placode (FC>2; FPKM>10) and more than 9000 active enhancers (Buzzi et al., 2022; Chen et al., 2017). We associated active enhancers to genes enriched in ear progenitors (see Materials and Methods; soft-annotation pipeline: https://github.com/Streit-lab/enhancer_annotation_and_motif_analysis) and screened them for Six1-binding motifs (Liu et al., 2012; Rauluseviciute et al., 2024; Sandelin et al., 2004) (Fig. S1; Table S1). This analysis resulted in 166 genes associated with 315 cis-regulatory elements (CREs) harbouring one or more Six1-binding sites. These genes are putative Six1 targets expressed in ear progenitor cells (Table S1).

**Fig. 1.**
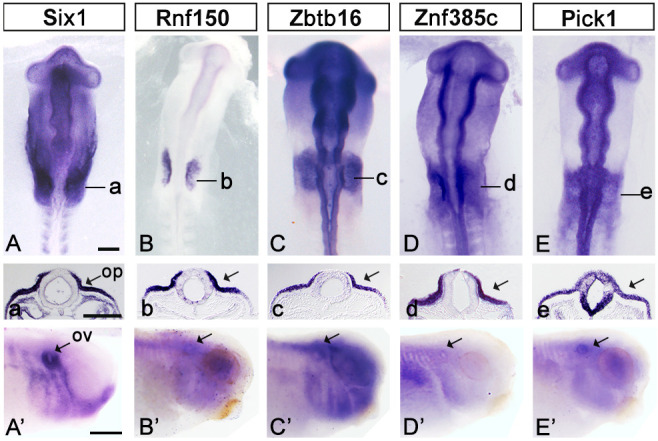
**Expression of putative Six1 targets.** (A-E′) *In situ* hybridisation for *Six1* (A,A′), *Rnf150* (B,B′), *Zbtb16* (C,C′), *Znf385c* (D,D′) and *Pick1* (E,E′) shows their expression in the chick otic placode (op) at HH10-11 (A-E) and in wild-type *Xenopus laevis* otic vesicles (ov) (A′-E′, arrows) at stages 30-32. Black lines in A-E indicate the level of sections shown in a-e. Arrows in a-e indicate the otic placode. Scale bars: 100 µm in A-E; 400 µm in A′-E′.

Based on their enrichment in otic progenitors and enhancer activity, we selected four genes as good candidate Six1 targets for further investigation. The two transcription factors *Zbtb16* and *Znf385c* occupy a central position in the ear gene regulatory network (Chen et al., 2017), and *Zbtb16* expression is reduced after Six1 knockdown in frog (Mehdizadeh et al., 2021). Likewise, *Rnf150*, which has been implicated in proliferation control (Deng et al., 2023), and *Pick1*, an adaptor protein controlling the subcellular localisation of membrane proteins (Li et al., 2016), have been proposed to be regulated by Six1 (Coppenrath et al., 2021). Whole-mount *in situ* hybridisation of 10-14 somite stage (ss) chick embryos reveals that all four transcripts are strongly expressed in the otic placode like *Six1* (Fig. 1A-E; see also Fig. S4A-D). Examining their expression in wild-type *Xenopus laevis* larvae (Fig. 1A′-E′) confirms that their expression is conserved in ear precursors, even across these very different vertebrate classes. Thus, these candidate Six1 targets are co-expressed with *Six1* in chick and frog.

### Six1 occupies target enhancers and Six1 motifs are required for their normal activity *in vivo*

**Fig. 2.**
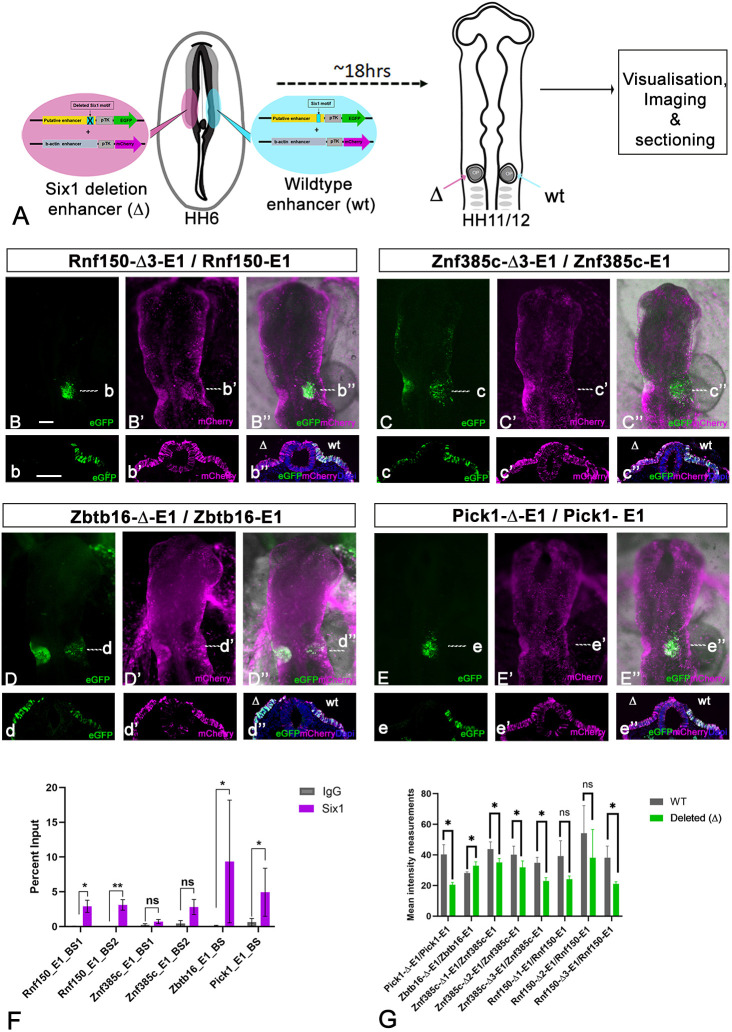
**Six1 occupies and regulates enhancer activity in the chick otic placode.** (A) Experimental strategy for bilateral electroporation of HH6/7 chick embryos with wild-type reporter constructs (right, blue) and eGFP reporters with the Six1 motif deleted (left, pink); each construct was co-electroporated with ubiquitously active mCherry. Embryos were assessed for enhancer activity at HH11-12. (B-B″) Rnf150-E1 activity in the otic placode with corresponding sections shown in b-b″; deletion of both Six1-binding sites (BSs) (Rnf150-Δ3-E1) leads to loss of enhancer activity (*n*=6). (C-C″) Znf385c-E1 activity in the otic placode with corresponding sections shown in c-c″; deletion of both Six1 motifs (Znf385c-Δ3-E1) leads to decreased enhancer activity (*n*=6). (D-D″) Zbtb16-E1 activity is moderate in the otic placode; corresponding sections shown in d-d″; Six1 motif deletion (Zbtb16-Δ-E1) leads to increased enhancer activity (*n*=5). (E-E″) Pick1-E1 activity in the otic placode with corresponding sections shown in e-e″; Six1 motif deletion (Pick1-Δ-E1) leads to reduced enhancer activity (*n*=4). Dashed white lines in B-E″ indicate the levels of sections shown in b-e″. Scale bars: 100 µm. (F) ChIP using Six1 and IgG control antibodies was performed on dissected chick otic placodes followed by qPCR for the enhancers indicated on the *x*-axis. *P≤0.05, **P≤0.01; ns, not significant; *P*≤0.09 for Znf385cBS1 and *P*≤0.08 for Znf385cBS2 (paired Student's *t*-test). Data are mean±s.e.m. (G) Quantification of GFP intensity as a proxy for enhancer activity in the otic placode before and after Six1 motif deletion. *P≤0.05 (paired Student's *t*-test). Data are mean±s.e.m.

The analysis above associated two CREs each to *Znf385c* and *Zbtb16*, but only one of each featured Six1-binding sites, while all three *Rnf150*-associated CREs and three out of four *Pick1*-associated CREs contained Six1 motifs. We selected one CRE for each gene for further analysis (Table S1). First, we assessed whether Six1 physically interacts with these CREs in ear progenitors. Otic placodes from ss10-12 chick embryos were dissected and processed for chromatin immunoprecipitation using Six1 and control IgG antibodies followed by qPCR for the relevant CREs. We find that, compared to control IgG, there is a significant increase in Six1 binding to CREs associated to *Rnf150*, *Zbtb16* and *Pick1*, while binding to the *Znf385c*-associated CRE is not significant (Fig. 2F). Thus, Six1 occupies CREs associated with genes enriched in inner ear progenitors.

To establish whether Six1 binding is crucial for normal CRE activity *in vivo*, we generated reporter constructs for *Rnf150-*E1-eGFP, *Zbtb16*-E1-eGFP, *Znf385c*-E1-eGFP and *Pick1*-E1-eGFP (see Table S1 for coordinates), as well as deletion constructs, in which the Six1 motif with the lowest *P*-value was removed (see Fig. S2 for Six1 motif position). Electroporation of wild-type constructs into HH6 chick embryos, together with ubiquitous mCherry shows that all are active in the otic placode (*Rnf150-*E1-eGFP 12/12, *Znf385c*-E1-eGFP 12/12, *Pick1*-E1-eGFP 9/9 and *Zbtb16*-E1-eGFP 10/10). To compare their activity in the same embryo, we electroporated HH6 stage chick embryos bilaterally with the wild-type and deletion constructs, together with ubiquitously expressed mCherry (Fig. 2A) and quantified eGFP fluorescence in the otic placode. The wild-type constructs *Rnf150-*E1-eGFP (12/12), *Znf385c*-E1-eGFP (17/17) and *Pick1*-E1-eGFP (4/4) show strong activity, while *Zbtb16*-E1-eGFP (5/5) shows moderate fluorescence (Fig. 2B-E″; Table S2). Deletion of the Six1 motif in *Pick1*-E1-eGFP (4/4; Fig. 2E,E″) and of both motifs in *Rnf150-*E1-eGFP (6/6; Fig. 2B,B″) leads to strong reduction of enhancer activity, while removal of only one Six1-binding site (BS) in *Rnf150-*E1-eGFP does not significantly reduce the fluorescence (BS1, *n*=4; BS2, *n*=2; Fig. S3A-B″; Table S2). *Znf385c*-E1-eGFP contains two Six1-binding sites; removal of either (BS1, 6/6; BS2, 6/6) or both (6/6) results in a strong reduction of enhancer activity (Fig. 2C,C″; Fig. S3C-C″; Table S2). These data show that the removal of the Six1 motif leads to a loss of normal enhancer activity, suggesting that Six1 may normally activate CREs associated with Rnf150, Pick1 and Znf385c. In contrast, deletion of the Six1 motif in *Zbtb16*-E1-eGFP increases enhancer activity (Fig. 2D-E″; 5/5; Table S2), indicating that Six1 may act as a repressor of this *Zbtb16* enhancer. Together, these experiments show that the CREs associated with *Rnf150*, *Zbtb16*, *Znf385c* and *Pick1* are active in inner ear precursors specifically, bound by Six1 except for *Znf385c-E1*, and that Six1 motifs are required for normal *in vivo* enhancer activity.

### Six1 controls the expression of its putative targets

**Fig. 3.**
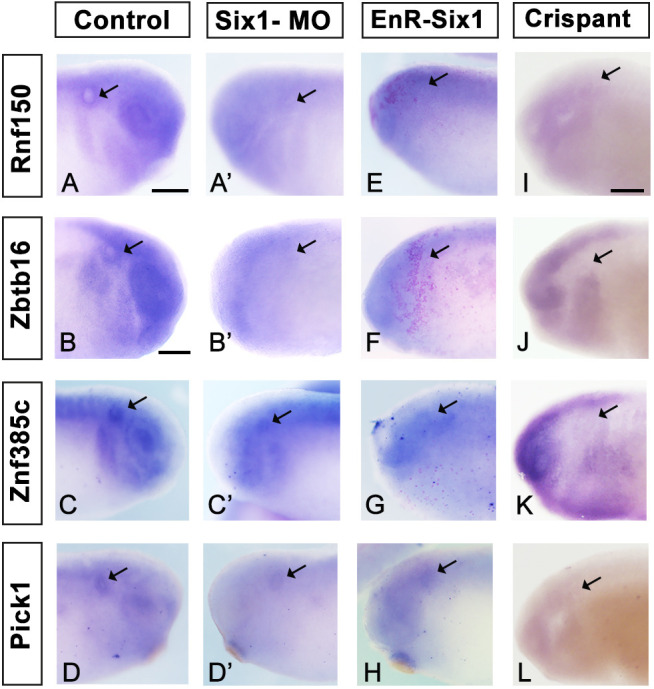
**Six1 is required for the expression of putative target genes.** (A-D′) *Rnf150* (A), *Zbtb16* (B), *Znf385c* (C) and *Pick1* (D) are expressed in the otic vesicle on the control side of *Xenopus laevis* larvae (A-D, arrows), but their expression is greatly reduced on the Six1 MO-injected side of the same embryo (A′-D′, arrows). (E-H) Injection of mRNA encoding EnR-Six1 into *Xenopus laevis* embryos leads to loss of the expression of all four genes; pink indicates lineage tracer demarking the injected side of the larva. (I-L) Six1 loss-of-function in *Xenopus tropicalis* F0 mutants created by CRISPR-Cas9 (Coppenrath et al., 2021) leads to loss of *Rnf150* (*n*=15) (I), *Zbtb16* (*n*=6) (J), *Znf385c* (*n*=12) (K) and *Pick1* (*n*=16) (L) expression in the otic vesicle (arrows). Scale bars: in A, 400 µm for A,C-H,A′,Cʹ,D′; in B, 400 µm in B,Bʹ; in I, 200 µm for I-L.

The above experiments suggest that the expression of *Rnf150*, *Zbtb16*, *Znf385c* and *Pick1* is regulated by Six1. To test this, we designed functional experiments in both *Xenopus* and chick. On one side of 8-cell *Xenopus laevis* embryos, we injected translation blocking morpholinos, previously tested for efficacy and specificity (Brugmann et al., 2004; Sullivan et al., 2019), into the dorsal animal and ventral animal blastomeres that together give rise to cranial structures (Moody and Kline, 1990). Larvae were harvested at stages 28-32 and processed for *in situ* hybridisation to assess changes in gene expression by comparing the staining intensity on the injected and uninjected side of the same embryo. In the majority, gene expression in the otic vesicles on the injected side was greatly reduced [Fig. 3A-D′; *Rnf150*: 76.2%, *n*=63; *Zbtb16*: 72.1%, *n*=61 (as reported by Mehdizadeh et al., 2021); *Znf385c*: 78.0%, *n*=50; *Pick1*: 72.7%, *n*=44]. In addition, otic vesicles were smaller in 17.8% of these morphants (*n*=218). To override normal Six1 function, we injected mRNA encoding a previously characterised Six1 constitutive repressor (*EnR-Six1*; Brugmann et al., 2004) unilaterally, as described above. This results in the reduction of *Rnf150* (67.3%, *n*=55), *Zbtb16* (91.9%, *n*=62), *Znf385c* (85.2%, *n*=54) and *Pick1* (78.4%, *n*=51) expression on the injected compared to the uninjected side of the same embryo (Fig. 3E-H). In 30.6% of these larvae (*n*=222), otic vesicles were smaller. Finally, we assessed gene expression levels in *Xenopus tropicalis* Six1 loss-of-function mutants in which a stop codon had been introduced upstream of the DNA-binding domain by CRISPR-Cas9 technology, leading to smaller otic vesicles (Coppenrath et al., 2021). In these larvae, the expression of all four genes was undetectable (Fig. 3I-L). Together, these three approaches demonstrate that Six1 is required for the expression of *Rnf150*, *Zbtb16*, *Znf385c* and *Pick1*, and suggest that it is an upstream regulator by either direct or indirect mechanisms.

**Fig. 4.**
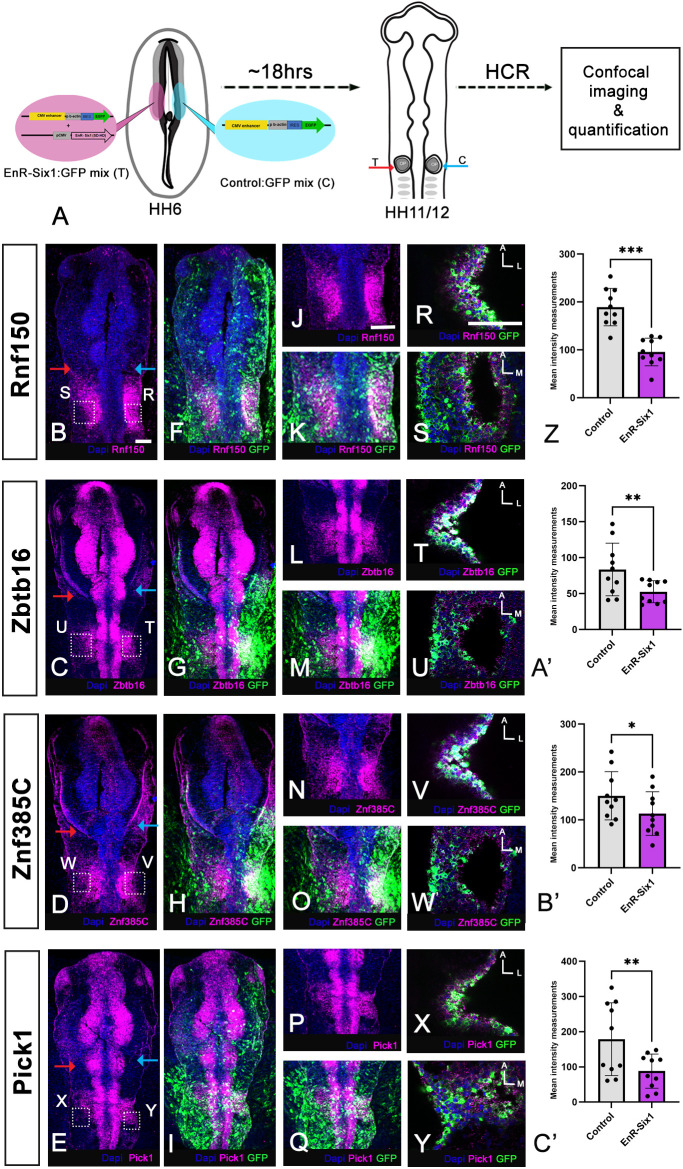
**Six1 regulates putative target gene expression in chick.** (A) Experimental design: EnR-Six1 and eGFP vectors were electroporated on one side of HH6/7 embryos (pink) and control eGFP vectors (blue) on the other. Embryos were grown until they reached HH11-12, and processed for HCR *in situ* hybridisation and imaging. (B-E) Whole-mount HCR *in situ* hybridisation for *Rnf150* (B), *Zbtb16* (C), *Znf385c* (D) and *Pick1* (E) in magenta; nuclei are stained using DAPI (blue). (F-I) Embryos in B-E with eGFP channel to visualise EnR-Six1 (left side of the embryo) and control GFP (right side of the embryo). The images shown in C,G and D,H represent the same embryo that was processed for double HCR *in situ* hybridisation. (J-Q) Higher magnifications of the otic placode territory of embryos shown in B-I. (R-Y) The outlined areas in B-E are imaged at a higher magnification and single confocal slices of these regions are shown. HCR signal is in magenta; GFP is in green. (Z-C′) Quantification of fluorescent gene expression signals on the control and experimental side. Dots indicate individual data points; data are mean±s.e.m.; *P<0.05, **P<0.01, ***P<0.001 (paired Student's *t*-test). Scale bars: 100 µm (bar in B applies to B-I; bar in J applies to J-Q; bar in R applies to R-Y).

Next, we performed complementary experiments in chick, allowing us to target ear progenitors at the time when they are specified. HH6 chick embryos were electroporated bilaterally with a mixture of EnR-Six1 and eGFP encoding plasmids on one side and control eGFP alone on the other (Fig. 4A). Embryos were harvested at the 12-14ss, processed for *in situ* hybridisation chain reaction (HCR) and imaged by confocal microscopy. As observed previously (Christophorou et al., 2009), EnR-Six1 leads to downregulation of the otic marker *Pax2* (see Fig. S4E for normal *Pax2* expression, Fig. S4F-J for EnR-Six1 experiment). Likewise, we find that *Rnf150*, *Zbtb16*, *Znf385c* and *Pick1* transcripts are absent or strongly reduced in cells electroporated with EnR-Six1/eGFP (*n*=10 per gene), while they express eGPF on the control side (Fig. 4B-Y). Quantification of the fluorescence intensity confirms that gene expression is reduced on the experimental side compared to the contralateral control side (Fig. 4Z-C′; Table S2). Together, these experiments provide evidence that Six1 regulates the expression of *Rnf150*, *Zbtb16*, *Znf385c* and *Pick1* in the otic placode and vesicle, in both chick and frog.

### Expression of putative Six1 targets human ear progenitors and their association with deafness loci

**Fig. 5.**
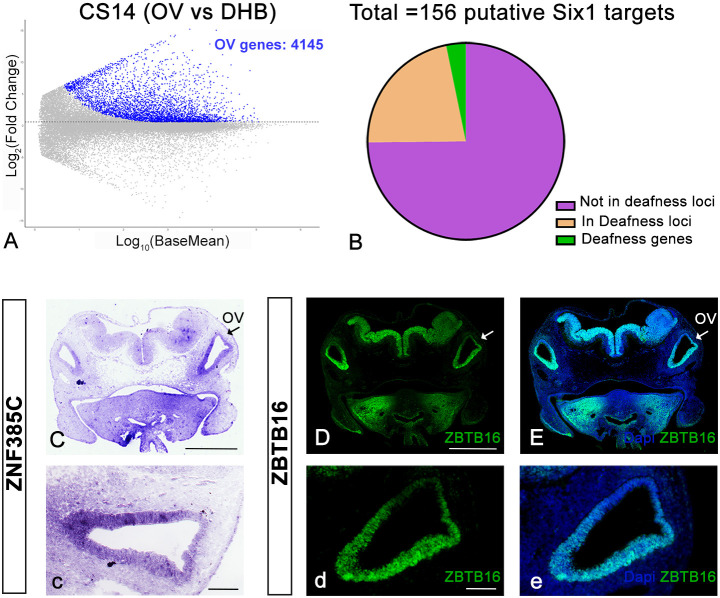
**Gene expression in human otic vesicles and candidate deafness genes.** (A) Volcano plot showing genes enriched in the otic vesicle (OV) of CS14 human embryos compared to the dorsal hindbrain (DHB). (B) Venn diagram showing putative Six1 targets located in human deafness loci. (C) *In situ* hybridisation for human *ZNF385C* on CS14 sections through the otic vesicle (OV; arrow). (c) Higher magnification of the OV shown in C. (D,E) Section through the OV of a human CS14 embryo stained using ZBTB16 antibodies (green); nuclei are visualised with DAPI (blue). (d,e) Higher magnification of the OVs shown in D and E. Scale bars: 500 µm in C,D; 100 µm in c,d.

To determine whether the newly identified candidate Six1 target genes represent candidate deafness genes in humans, we first performed RNA-sequencing (RNAseq) from dissected otic vesicles and from the adjacent dorsal hindbrain from CS13 and CS14 human embryos. Overall, the transcriptome of the ear tissues differs from that of the hindbrain (Fig. S5A). Differential gene expression analysis identifies 4145 and 3508 transcripts enriched in otic vesicles compared to dorsal hindbrain at CS13 and CS14, respectively (Log2FC>1.5, adjusted *P* value <0.1; Table S3; Fig. 5D′). We identified 156 human orthologs of the 166 putative chick Six1 targets (Table S3). Of these, 121 genes are enriched in human otic vesicles when compared to hindbrain at CS13 and/or CS14, while the remaining 35 show higher expression levels in the hindbrain (Table S3; Fig. S5C). Next, we verified the expression of the two transcription factors *Znf385c* and *Zbtb16* in sections of human CS14 embryos using *in situ* hybridisation and immunostaining, respectively. We find that Zbtb16 protein is strongly expressed throughout the otic vesicle, except its most dorsomedial tip (Fig. 4E′,F′). Likewise, *Znf385c* transcripts are strongly expressed in the ventral otic vesicle, with weaker expression in its most dorsal region (Fig. 4G′).

Next, we assessed whether the enhancers for *Rnf150*, *Zbtb16*, *Znf385c* and *Pick1* identified in chick are conserved in humans. We find that this is indeed the case for *ZBTB16*, *ZNF385C* and *PICK1*, while there is no sequence conservation for the *RNF150*-associated CRE (Fig. S2A′-D′). Six1-binding site analysis of these putative human enhancers reveals that, while the putative CRE associated with *ZBTB16* does not contain a Six1 motif, one motif was found in the putative *PICK1* enhancer and two in the CRE associated with *ZNF385C* (Table S4)*.* Together, these experiments show that most putative Six1 targets identified in chick are also expressed in the human otic vesicle and that some enhancers harbouring the Six1 motif are conserved in humans. Therefore, the candidate Six1 targets identified in chick are also candidates to mediate its function in human ear progenitors.

Finally, we assessed whether the human putative Six1 targets represent new candidate deafness genes. We retrieved the coordinates of disease-associated loci from OMIM and extracted 235 known deafness loci, including syndromic and non-syndromic deafness (Tables S3, S4). For 185 loci, the causative gene has been identified, including the candidate Six1 targets *SIX1*, *JAG1*, *LMX1A*, *SPATA5* and *HOMER2* (Fig. 4H′; Table S3). For the remaining 50, the causative gene(s) remain unknown. We extracted all genes located within these 50 loci (5023 genes in total) and cross-referenced them to the human putative SIX1 targets genes. Of 156 targets, 36 (23%) are indeed located within a known deafness locus (Fig. 4H′; Fig. S5B). Among these genes are those encoding cell-adhesion molecules, enzymes and modulators of the cytoskeleton (Fig. S5B, Table S3) that may play various roles in ear development. Interestingly, putative Six1 targets also encompass components of signalling pathways known to regulate ear development. These include: KREMEN1, a receptor for the Wnt antagonist Dkk; the G-protein coupled receptor LGR4, which binds R-spondin and activates Wnt signalling (Glinka et al., 2011); and the mitogen-activated protein kinase 8 interacting protein 1, also known as Jip1, which is activated by Wnt signalling (Cai et al., 2002). Likewise, BMP7 expressed in different cell types throughout ear development (Groves and Bronner-Fraser, 2000; Oh et al., 1996) is a candidate Six1 target, and has been implicated in cochleovestibular ganglion formation and in establishing tonotopy along the cochlea (Fantetti and Fekete, 2012; Mann et al., 2014). Together, our findings implicate these newly identified putative Six1 targets as new candidate deafness genes.

Among the putative human Six1 targets are also the metallophosphoesterase MPPED2, which has been associated with chronic kidney disease (Pattaro et al., 2012; Zhong et al., 2023), and the serine/threonine kinase 39 (STK39), also called SPAK. STK39 is involved in potassium homeostasis (Alessi et al., 2014), which is important for normal function of both the ear and the kidney. These genes may therefore be two new BOR candidate genes.

## DISCUSSION

The transcription factor Six1 and its co-factor Eya1 control the formation of many organ systems, including sense organs, kidney, thymus and muscle, where they regulate cell fate specification and proliferation (Brugmann et al., 2004; Chen et al., 2009; Christophorou et al., 2009; Grifone et al., 2005; Ikeda et al., 2007; Laclef et al., 2003a,b; Ozaki et al., 2004; Xu et al., 1999, 2003; Zheng et al., 2003; Zou et al., 2008, 2006a,b, 2004). However, the molecular pathways downstream of these factors are poorly understood. Here, we identify previously unreported candidate Six1 target genes in chick, frog and human ear progenitor cells. We find that about one quarter of these genes are associated with human deafness loci where the causative gene has not yet been identified, suggesting that they represent previously unidentified candidate deafness genes.

Ear progenitors are set aside early in development in a patch of ectoderm next to the hindbrain, known as the otic placode (Chen and Streit, 2013; Ohyama et al., 2007; Wu and Kelley, 2012). Subsequently, the placode invaginates and the vesicle is transformed into the complex 3D structure of the adult inner ear, while simultaneously generating many specialised cell types, including neurons, sensory hair cells and others. In chick, the activation of Six1 target genes is required for placode formation at early stages (Christophorou et al., 2009). Here, we have identified 166 putative direct Six1 targets by interrogating active otic enhancers for the presence of Six1-binding motifs. Selecting enhancers associated with four candidates (*Rnf150*, *Zbtb16*, *Znf385c* and *Pick1*), we show that Six1 motifs are essential for normal enhancer activity *in vivo* and that Six1 occupies three of these enhancers in ear progenitors. Finally, functional experiments in chick and frog show that the four transcripts are indeed regulated by Six1.

Our enhancer analysis indicates that Six1 may be a direct activator of the CREs associated with *Rnf150*, *Pick1* and *Znf385c*: deletion of the Six1 motif leads to reduction or loss of enhancer activity in reporter assays. Accordingly, knockdown or loss of Six1 in *Xenopus* shows that their expression requires Six1 function. Together, these data suggest that Six1 directly activates the expression of these genes. In contrast, the regulatory relationship between Six1 and Zbtb16 appears to be more complex. Six1 motif deletion in the *Zbtb16*-associated CRE increases reporter activity, suggesting that Six1 binding mediates repression through this enhancer. However, functional experiments in *Xenopus* reveal that Six1 is required for *Zbtb16* expression. There are different scenarios that may explain this apparent discrepancy. It is possible that the enhancer identified does not regulate *Zbtb16*, despite its close association, or that multiple enhancers control its expression, of which we have examined only one, and that the balance of their activity ultimately determines *Zbtb16* expression levels. On the other hand, Six1 may regulate an upstream activator of *Zbtb16* that is crucial for the onset of its expression, while later being involved in *Zbtb16* repression via the identified enhancer. For example, we have previously shown that the transcription factor Pax2 is required for *Zbtb16* expression (Chen et al., 2017) and that, in turn, *Pax2* is regulated by Six1 (Christophorou et al., 2009). This example highlights the complexity of the gene regulatory network controlling otic placode specification; unravelling the precise topography of this network requires further experiments, including those aimed at establishing the interaction of enhancers and promoters.

So far, Six1 target genes have only been identified by large scale screens in *Drosophila* (Jusiak et al., 2014), in developing mouse hair cells (Li et al., 2020), and in mouse and human nephron progenitors (O'Brien et al., 2016). These have identified transcriptional regulators involved in cell fate decisions, signalling pathway components and cell cycle control genes, some of which overlap with the genes identified here. Of the 166 putative chick Six1 targets, more than half (94) are also associated with Six1-bound putative enhancer regions in mouse hair cells (Li et al., 2020), suggesting that they are indeed bona fide Six1 targets. Our analysis reveals that known regulators of ear development, such as Gbx2 (Lin et al., 2005; Steventon et al., 2012), Lmx1a (Chen et al., 2017; Mann et al., 2017), Sox10 (Breuskin et al., 2009; Szeto et al., 2022; Taylor and Labonne, 2005), Irx1 (Sullivan et al., 2019) and Six1 itself, may directly be regulated by Six1. These transcription factors occupy key positions in the otic gene regulatory network (Buzzi et al., 2022; Chen et al., 2017) and our findings place Six1 at the top of the genetic hierarchy that controls ear progenitor specification. In addition, we identify components of the BMP, Wnt and Shh pathways, which have been implicated in otic induction and patterning (Basch et al., 2016; Bok et al., 2007; Groves and Fekete, 2012; Wu and Kelley, 2012), as candidate direct Six1 targets. Thus, Six1 not only controls the transcriptional programme for ear formation, but also coordinates signalling events between different ear compartments and/or cell types, and the cell populations surrounding the developing ear. This scenario resembles its role in kidney development. Here, Six1 is required for the specification of nephron progenitors and maintenance of the progenitor state, as well as controlling signalling to the surrounding metanephric mesenchyme (Kobayashi et al., 2007; Xu et al., 2022, 2003).

In the context of human development, we provide the first molecular characterisation of otic vesicle stages. We find that, when compared to the adjacent dorsal hindbrain, 4145 transcripts are enriched in the otic vesicle at early CS13, while 3508 are enriched at CS14. Among these are components of many known signalling pathways and transcriptional regulators of ear development, although further studies are needed to define their spatial expression patterns. Importantly, of the putative 166 Six1 targets identified in chick, 155 are also expressed in the human otic vesicle. Assessing the conservation of enhancers for *RNF150*, *ZBTB16*, *ZNF385C* and *PICK1*, we find that three are indeed conserved, suggesting that these genes may be direct SIX1 targets in humans. However, the lack of regulatory data from human developing ears does not allow us to assess whether these or other enhancers are active.

Interestingly, about one quarter of the putative human Six1 targets are associated with human deafness loci where the causative gene has not been identified. Many of these contain multiple genes and our analysis will help to prioritise putative Six1 targets for functional evaluation. Wnt-related proteins feature prominently in our newly identified candidate deafness genes. LGR4 is a receptor involved in activation of the Wnt pathway. In humans, its loss leads to reduced Wnt activity and to severe birth defects, including hypoaldosteronism, nail and stature defects, and deafness (Lucas et al., 2023). Activated by Wnt signalling, the mitogen-activated protein kinase 8 interacting protein 1, also known as Jip1, phosphorylates Pax2 to increase its transcriptional activity (Cai et al., 2002). In turn, Pax2 is a key regulator of inner ear progenitor specification and proliferation, as well as cochlear development (Burton et al., 2004; Christophorou et al., 2010; Freter et al., 2012). It is therefore possible that Jip1 mutations in humans might affect Pax2 function and consequently lead to deafness. Finally, KREMEN1 is a receptor for the Wnt antagonist DKK1 and together they block canonical Wnt signalling. *KREMEN1* is expressed in early ear progenitors (Lleras-Forero et al., 2013; this study) and inhibits hair cell specification in zebrafish (Megerson et al., 2024). A role for DKK1 in ear development has not been reported. With Wnt signalling playing multiple roles in ear formation (Basch et al., 2016; Bok et al., 2007; Groves and Fekete, 2012; Wu and Kelley, 2012), Wnt pathway members should be prioritised as candidate deafness genes for future research.

Among the birth defects associated with hearing loss, BOS and BOR syndromes represent the second most common (Moody et al., 2015; Smith, 2018) but causative mutations have been identified in only about 50% of patients. Here, we have discovered two putative Six1 targets as new BOR/BOS candidates. The metallophosphoesterase MPPED2 is associated with chronic kidney disease (Pattaro et al., 2012; Zhong et al., 2023). It is expressed in ear progenitors (this study) as well as in the mesenchyme surrounding the ear (van der Valk et al., 2023); however, a role in ear development has not been reported. Serine/threonine kinase 39 (STK39), also called SPAK, is part of the WNK-SPAK/OSR1 complex involved in potassium homeostasis (Alessi et al., 2014). In the ear, maintenance of appropriate potassium concentration of the endolymph is crucial for normal hearing (Locher et al., 2015; Zdebik et al., 2009), while the kidney plays a key role in potassium homeostasis (Wieërs et al., 2022). How SPAK activity may regulate ear development remains to be explored. Thus, both of these genes should be prioritised for screening as new potential BOR candidates.

### Conclusions

In summary, the identification of new candidate Six1 target genes not only provides new information on the mechanisms of inner ear progenitor information during development, but has also allowed us to propose new candidate genes for BOR/BOS and other forms of deafness. Future studies will need to clarify their functional role within the gene network controlling ear formation. Importantly, we also describe new regulatory regions as likely Six1 targets in chick. While some of these CREs are conserved in humans, for many the corresponding regions in the human ear are unknown. Mutations in such enhancers are likely to cause hearing loss, as they will affect gene expression in the ear. Future research will need to employ large-scale approaches to find active enhancers in the developing human inner ear.

## MATERIALS AND METHODS

### Animal handling procedures

#### Chicken embryos and culturing

Chicken embryos used in this study are younger than 12 days, hence are not regulated by Animal Scientific Procedures Act 1986. All procedures were carried out according to the institutional guidelines. Fertilised hens' eggs were obtained from Winter Egg or Henry & Steward Farms (UK) and incubated in a humid incubator at 38°C to reach the desired stage, based on Hamburger and Hamilton (HH) (Hamburger and Hamilton, 1951). Head fold stage (HH5/6) embryos were cultured using the Early Chick (EC) culturing method as described previously (Chapman et al., 2001) and used for electroporation.

#### *Xenopus* embryos and microinjections

Fertilised *Xenopus laevis* eggs were obtained by gonadotropin-induced natural mating of wild-type, outbred adult frogs that were obtained from Nasco Education (Fort Atkinson, WI) and housed at The George Washington University. All procedures and experiments involving *Xenopus laevis* were approved by the Institutional Animal Care and Use Committee (IACUC) at The George Washington University (A2022-020). Embryos were selected at the two-cell stage if the first cleavage furrow bisected the lightly pigmented region of the animal hemisphere to identify the dorsal-ventral axis accurately (Klein, 1987; Miyata et al., 1987). When they reached the 8-cell stage, the dorsal animal and the ventral animal blastomeres that give rise to the cranial tissues (Moody and Kline, 1990) were each microinjected on one side of the embryo with 1 nl of either mRNAs or antisense morpholino oligonucleotides (MOs), together with mRNA encoding nuclear-localised β-galactosidase (nβgal) for lineage tracing (Moody, 2018a,b). Six1 loss-of-function F0 mutants in the diploid *Xenopus tropicalis* were produced at the National Xenopus Resource, as previously described (Coppenrath et al., 2021). Using CRISPR-Cas9 technology, a stop codon was introduced upstream of the Six1 DNA-binding domain.

#### Human embryonic tissue and processing

Pre-staged human embryo samples were obtained from the Human Developmental Biology Resource (HDBR) tissue bank for performing bulk RNAseq, *in situ* hybridisation and immunocytochemistry, respectively. Sample ID 14479 (200322), 15409 and 15441 were staged as CS13, CS14 and CS15, respectively. However, based on morphological features observed while dissecting and sectioning, and comparing with the 3D reconstructions of human development (de Bakker et al., 2016), these samples were re-staged as follows: ID 15409, CS13; 15441 and 14479 (200322), CS14. Ethics approval was obtained from the HDBR.

Otic vesicles (left and right pooled) and dorsal hindbrain tissues from a single embryo were dissected for each biological replicate from sample ID: 15409 and 15441. Dissected tissues were collected into low-binding tubes with 40 µl NEB protection buffer (T2010) on ice and frozen immediately at −80°C. All samples were stored at −80°C until processing for RNA sequencing.

Fixed tissue received from the HDBR [sample ID: 14479 (200322)] was dehydrated in increasing concentrations of ethanol (25%, 50%, 70% and 100% diluted in H_2_O) at room temperature. To allow wax penetration, the sample was incubated and washed in Xylene at room temperature until the tissue became transparent. Furthermore, three washes in wax were performed at 60°C for 30 min to 1 h each, and the specimen was embedded and allowed to set overnight at 4°C. The blocks were trimmed with a razor blade and sectioned with a Leica RM2245 microtome at 12 μm, mounted on slides (Slide Superfrost Plus, SLS) and stored until further processing.

### Chicken experimental procedures

#### Enhancer cloning and bi-lateral electroporation

Chicken genomic DNA was used to amplify putative enhancer elements and cloned into modified pTK-EGFP reporter vectors after digestion with XcmI (Chen and Streit, 2015). To delete a SIX1-binding site (BS) of interest, primers were designed complementary to the regions flanking the area of SIX1-BS within each enhancer. The wild-type enhancer reporter constructs were used as a template to amplify enhancer regions without SIX1 BS. The amplified DNA fragment was cloned back into an empty pTK-EGFP reporter vector. Primers used for cloning wild-type and deletion enhancers constructs are listed in Table S1. All sequences were verified by Sanger sequencing.

Bilateral electroporation was used to transfect developing otic placodes with various DNA plasmids (Table S5). For electroporation, EC cultured embryos were transferred (ventral side facing up) into an electroporation chamber (2×2 mm platinum negative electrode) containing Tyrode's solution. Electroporation mix (2-2.5 μg/μl report constructs/EnR-Six1 overexpression construct+0.5-1.5 μg/μl pCAB-IRES-GFP/RFP+5% sucrose in H2O with 0.1% Fast Green for visualisation) was injected between the vitelline membrane and the ectoderm using fine capillary glass needles; two consecutive injections, using two sperate capillary glass needles, were performed on each side of the same embryo. Following injection, a positive platinum electrode (2×1 mm) was positioned above the target area and five pulses of 5 V were applied for 50 ms at 750 ms intervals using an Intracell TSS20 OVODYNE pulse generator. Embryos were incubated in a Petri dish containing 1 ml of thin albumen in a humid chamber at 38°C until they reached the desired stage. Embryos were imaged using a Zeiss Axiovert 200M coupled to a Hamamatsu digital camera (c4742-95), and/or an Olympus SZX12 dissecting microscope and a Retiga 2000R digital camera. Embryos were later separated from the filter paper and vitelline membrane for further processing.

#### Whole-mount immunostaining and cryosectioning

Well-electroporated embryos were processed for whole-mount immunostaining against GFP (1:1000; Molecular Probes, A11120), and mCherry (Abcam, ab167453; 1:200) as described previously (Buzzi et al., 2022). Whole-mount images were acquired using an Olympus SZX12 with a Retiga2000R camera and Q-Capture Pro7 software (Fig. 2 and Fig. S3). Embryos were embedded in gelatin, as previously described (Stern and Holland, 1993). Using a Bright OTF5000 cryostat, 15-20 μm sections were transferred on pre-coated gelatin slides and mounted with Mowiol 4-88 (Sigma Aldrich, 81381). The sections were visualised under a Zeiss Axiovert 200 M microscope and photographed with a Hamamatsu C4742-95 camera using OCULAR software.

#### Whole-mount DIG *in situ* hybridisation and hybridisation chain reaction *in situ* hybridisation

Whole-mount *in situ* hybridisation (WISH) was performed as described previously (Streit and Stern, 2001) and viewed with an Olympus SZX12 microscope. Digoxigenin-labelled antisense RNA probes were generated using DNA plasmids as a template and transcribed using specific RNA polymerases (see Table S6). Paraffin wax-embedded WISH embryos were sectioned at 12 μm and viewed with a Zeiss Axiovert 200 M microscope. Whole-mount embryo and section images were captured using a Retiga2000R camera and Q-Capture Pro7 software.

HCR v3 was performed following the manufacturer's protocol (Molecular Technologies) as described previously (Buzzi et al., 2022). Whole-mount images were captured using Zeiss LSM850 confocal microscopy with a 10× or 40× objective and are displayed as maximum intensity projections of *z*-stacks (step size=10 μm). Images in Fig. 4R-Y are single confocal slices from 40× images.

#### Image quantification and statistical analysis

For quantification, the whole-mount HCR images were captured using 40× objective with a digital zoom of 0.6× and optically sectioned with a step size of 5 μm. Only regions of the otic placode were subsetted and electroporated cells (identified by GFP signal) were used for quantification analysis. Regions of interest (ROIs) for each *z*-slice were defined using auto-thresholding method (IsoData dark) in the GFP channel. Mean fluorescence intensity within each ROI for each slice was measured and summed to calculate the expression levels for each transcript in each embryo. The intensity measurement calculations were semi-automated using a macros script in ImageJ. To test for significant difference, the summed intensity measurements for each transcript in control and EnRSix1 transfected otic placodes were compared using a paired Student's *t*-test. Statistical significance was determined at a significance level of α=0.05.

To quantify changes in enhancer activity after Six1 motif deletion, we used bilaterally electroporated embryos, immunostained for anti-GFP and anti-mCherry, and whole-mount imaged (as described above). Only the region of the otic placode was selected and electroporated cells (identified by mCherry signal) were used for quantification analysis. ROIs were defined using am auto-thresholding method (percentile) in the mCherry channel. Mean GFP fluorescence intensity within each ROI for the otic placode was measured using ImageJ as a readout to calculate enhancer activity. Mean intensity measurements for wild-type and mutated enhancer transfected otic placodes were compared using a ratio paired Student's *t*-test (α=0.05, one-tailed).

### Chromatin immunoprecipitation and quantitative PCR

Otic placodes from HH11/12 stage embryos were micro-dissected and processed for chromatin immunoprecipitation (ChIP) as described previously (Tambalo et al., 2020). A total of 100 otic placodes were pooled for each biological replicate. ChIP was performed using the following antibodies: anti-IgG (Millipore CA92590; 5 μg per IP) and anti-Six1 [Sigma HPA001893; 5 μg per IP; (Li et al., 2020)]. ChIP for Six1 and control (IgG) was processed for three biological replicates independently. ChIPed chromatin was purified using phenol: chloroform: isoamyl alcohol and resuspended in 30 μl of water. Ct values were obtained for each IP using quantitative PCR (qPCR) and normalised against input chromatin. The primer sequences used are provided in Table S1. To assess the enrichment of Six1 in the selected enhancer regions, the ratio of Six1 ChIP signal (percent input) to IgG ChIP signal was compared for each region, and a ratio paired student *t*-test was performed. Statistical significance was determined at a significance level of α=0.05.

### Motif discovery and annotation of putative enhancers

H3K27Ac ChIPseq and ATACseq data were taken from Buzzi and colleagues (Buzzi et al., 2022). ATAC data were re-aligned using the NF-core ATACseq pipeline (version: 1.2.0) using default parameters to obtain broad peaks. ATAC and H3K27Ac peaks were intersected using BEDTools suite (v2.30.0) (Quinlan and Hall, 2010) and annotated using HOMER (Heinz et al., 2010) available in galaxy web server (UseGalaxy.eu). Peaks annotated to promoters and exons were excluded to focus on putative enhancers. Putative enhancers were then processed through our custom enhancer annotation and motif analysis Nextflow pipeline (version: 1.0), which is publicly available at: https://github.com/Streit-lab/enhancer_annotation_and_motif_analysis. In brief, putative enhancers were assigned to nearby transcriptional start sites (TSSs) within an 35 kb window. Otic placode-enriched genes were taken from Chen and colleagues (Chen et al., 2017) using the comparison of ss 8/9 placodes to ss3 whole embryo (foldchange >2 and FPKM>10). Their coordinates were extracted using Biomart and converted to Galgal6 coordinates using UCSC liftOver tool. Putative otic enhancers were obtained by filtering peaks annotated to these otic enriched genes. Putative otic enhancers were then screened for Six1 motifs using the MEME suite package FIMO. Motifs hits were filtered based on a *P*-value threshold of 0.001. Three different position weight matrices (Liu et al., 2012; Rauluseviciute et al., 2024; Sandelin et al., 2004) were used for binding site analysis (Table S1).

### *Xenopus* experimental procedures

#### *In vitro* synthesis of mRNAs and antisense RNA probes

mRNAs encoding *Xenopus laevis* EnR-Six1 and nuclear localised βgalactosidase (nβgal; lineage tracer) were synthesised *in vitro* (mMessage mMachine kit, Ambion). The repressive EnR-Six1 construct was generated by ligating the N-terminal Six domain+homeodomain of *Xenopus laevis* Six1 downstream of the *Drosophila* Engrailed repressor domain (Brugmann et al., 2004), according to the methods of Pohl and Knochel (Pohl and Knochel, 2001). Plasmids encoding Pick1, Rnf150, Zbtb16 and Znf385c were purchased from Open Biosystems/Dharmacon (Table S7), subcloned into Gateway vectors as part of the ORFeome project (Grant et al., 2015), and are now available from Horizon Discovery in their *Xenopus* Collection (https://horizondiscovery.com/en/non-mammalian-research-tools/products/xenopus-collection). Antisense RNA probes for *in situ* hybridisation were synthesised *in vitro* (MEGAscript kit; Ambion), as previously described (Sullivan et al., 2001).

#### Antisense oligonucleotides

To knock down the endogenous level of Six1 protein, two translation-blocking antisense morpholino oligonucleotides that bind to *Xenopus laevis* Six1 mRNA (Six1-MO) were co-injected at equimolar concentrations (9 ng/blastomere). Their specificity and efficacy have been previously validated both biochemically and *in vivo* (Brugmann et al., 2004; Sullivan et al., 2019).

#### Fixation, histochemistry and *in situ* hybridisation

Wild-type, microinjected and mutant embryos were cultured to neural tube (stage 28-32) stages (Nieuwkoop and Faber, 1956), fixed in 4% paraformaldehyde (in 0.1 M MOPS, 2 mM EGTA magnesium and 1 mM MgSO_4_ at pH 7.4), stained for β-Gal histochemistry if injected with mRNAs, and processed for *in situ* hybridisation as previously described (Sullivan et al., 2001). Each experiment was repeated in two to five independent trials with different sets of parents.

### Human experimental procedures

#### Bulk RNA sequencing and data analysis

RNA purification, library preparation and RNA sequencing were carried out at Novogene (www.novogen.com). Generally, tissues were processed using the NEB Monarch kit (T2010) for RNA isolation. Messenger RNA was purified from total RNA using poly-T oligo-attached magnetic beads. After fragmentation, the first strand cDNA was synthesised using random hexamer primers followed by the second-strand cDNA synthesis. The library was ready after end repair, A-tailing, adapter ligation, size selection, amplification and purification. RNA and cDNA quality were checked with Qubit and real-time PCR for quantification and with a bioanalyzer for size distribution detection. Quantified libraries were pooled and sequenced at a depth of 1 million reads on an Illumina HiSeq 4000 (150 bp paired-end reads).

Following standard quality control, paired-end reads were aligned to human GRCh38 genome. Alignment was performed using HiSAT2 with the default parameters in Galaxy version 2.1.0 available on the galaxy web server (usegalaxy.org) (The Galaxy Community, 2024). To facilitate quantitative gene expression analysis, aligned reads for each sample were counted using featureCounts (Liao et al., 2014). Differential gene expression analysis was carried out with DESeq2 package in R (Love et al., 2014). Adjusted *P* values were calculated using the default DESeq2 multiple test correction (Benjamini–Hochberg). Differentially expressed transcripts were determined by an absolute log2 fold-change >1.5 and adjusted *P* value<0.1.

Human orthologs of Six1 targets identified in chick and putative Six1 targets in deafness loci were used to perform hierarchical clustering (Pearson's correlation) to group genes with similar expression profile. To visualise the expression profiles of genes of interest in different tissues and stages, averaged normalised counts generated by DESeq2 were transformed into row z-scores with heatmap.2, and corresponding heatmaps were generated using gplot within R (https://www.r-project.org/; https://cran.r-project.org/package=gplots).

#### *In situ* hybridisation on paraffin sections

Human ZNF385C DNA inserted in pExpress-1 vector backbone was purchased from Source Bioscience. DNA template to generate digoxigenin-labelled antisense probe was amplified by PCR using M13 forward (M13F: 5′-GTAAAACGACGGCCAGTG-3′) and M13 reverse (M13R: 5′-GGAAACAGCTATGACCAT G-3′) primers and transcribed using T7 polymerase.

Paraffin wax-embedded sections were dewaxed in three 10 min washes in xylene and rehydrated using a descending series of ethanol (100%, 70%, 50% and 25% in DEPC-treated water) for 5 min each, followed by two 5 min washes in DEPC-PBS. Sections were fixed in 4% PFA in DEPC-PBS for 20 min, followed by two 5 min washes with DEPC-PBS. To permeabilise the tissue, sections were treated with 20 µg/ml proteinase K diluted in DEPC-PBS for 8 min, followed by tissue refixation using 4% PFA in PBS. To prevent non-specific background, sections were washed in freshly made acetylation solution (0.25% acetic anhydride and 0.1 M triethanolamine in DEPC PBS) for 10 min, followed by two 5 min washes with DEPC-PBS. Sections were then dehydrated in an ascending ethanol series, as described above, and air-dried.

DIG labelled anti-sense riboprobe (0.5-1 µg/ml) diluted in hybridisation buffer [50% deionised formamide (Sigma), 20 mM Tris-DEPC (pH 7.5), 0.3 M NaCl-DEPC, 5 mM EDTA-DEPC, 1× Denhardt's (Denhardt's Solution 50×, Sigma) and 10% dextran sulphate sodium salt (Sigma) in DEPC-H_2_O] was denatured at 85°C for 2 min and chilled on ice for 3 min. Denatured probes were mixed with 1 µl/ml of RNasin Ribonuclease Inhibitor (Promega) and 0.5 mg/ml of tRNA (diluted in hybridisation buffer; tRNA from brewer's yeast, 10109517001 Roche); 350 µl of probe solution was gently applied onto the sections of each slide. Slides were hybridised overnight at 65°C in a humidified chamber (humidifying solution: 50% formamide and 2×SSC in DEPC-H_2_O).

After hybridisation, the sections were washed with 2×SSC solution (pH=7.5) for 20 min to remove probe solution. Sections were further washed with post-hybridisation solution (50% formamide and 2×SSC in H_2_O) and 2×SSC for 30 min each at 65°C, followed by two washes in 0.2×SSC at 65°C and another in 0.2×SSC at room temperature for 30 min each. Sections were briefly rinsed in TBS [0.1 M Tris (pH 7.5) and 0.15 M NaCl in H_2_O] and then incubated in blocking buffer (10% heat-inactivated sheep serum+0.05% Tween-20 in TBS) in a water-humidified chamber for 2 h. Anti-DIG antibody was then applied to the sections for overnight incubation at 4°C.

To stain the sections, slides were washed twice in NTMT (0.1 M Tris, 0.1 M NaCl, 0.05 M MgCl_2_ and 0.05% Tween-20 in H_2_O) for 10 min and BM Purple was added per slide. After the colour reaction had fully developed, it was stopped by two 5 min washes of PBS. Sections were mounted using the aqueous mounting medium Aquatex and imaged with Zeiss ApoTome.2 coupled to an Axiocam 503 colour camera.

#### Immunofluorescence histochemistry on paraffin wax-embedded sections

Tyramide signal amplification (TSA) Plus cyanine 5 fluorescein system kit (AKOYA Bioscience) was used in this protocol to amplify antibody fluorescent signal. Paraffin wax-embedded sections were dewaxed in Xylene with three 10 min washes, followed by tissue rehydration in a decreasing ethanol series (100%, 75%, 50% and 25% diluted in H_2_O) and two 5 min washes in PBS.

Sections were treated with 3% Tween-20 in PBS for 30 min at room temperature and washed twice in PBS for 10 min. Antigen retrieval was performed using freshly made 10 mM sodium citrate buffer at pH 6 [Trisodium citrate dihydrate (Sigma) in H_2_O] in a pressure cooker, incubated at 95°C in a water bath for 40 min and cooled to room temperature for 10 min before washing twice with TBST (TBS with 0.025% Tween-20) for 10 min. Sections were then blocked for 1 h at room temperature in blocking buffer [0.5% blocking reagent (from the TSA kit), 10% heat-inactivated goat serum and TBST] and incubated with anti-ZBTB16 antibody (1:200; Thermo Fisher Scientific, 39987) overnight at 4°C.

Sections were washed four times in TBST for 10 min each and before incubating in goat anti-mouse biotinylated secondary antibody (1:600; Vector Laboratories, BA-9200) for 2 h in room temperature. Afterwards, slides were washed three times with TBST for 10 min and incubated in DAPI (0.1 μg/ml in blocking buffer) for 15 min. followed by another two 10 min TBST washes.

Tissues were further treated with 150 µl of 50 µg/ml Streptavidin HRP solution (Abcam) per slide for 30 min at room temperature and washed three times for 10 min with TBST. Finally, Tissues were incubated with Tyramide signal amplification buffer [0.1 M borate buffer pH=8.5 (Sigma) and 0.003% H_2_O_2_ in H_2_O] for 10 min at room temperature and washed three times with TBST for 10 min each. Slides were mounted by Fluoromount-G (Southernbiotech) and imaged using a Zeiss ApoTome.2 coupled with an Axiocam 503 colour camera.

#### Deafness loci analysis

Genes and loci associated with deafness (syndromic and non-syndromic) were subset from the OMIM database (https://omim.org/). Genomic coordinates for all annotated human genes (assembly: hg38) were extracted from the UCSC table browser (Nassar et al., 2023). For deafness loci without known candidate genes, the genomic coordinates were extracted and extended 100 bp upstream and downstream using the slop function from the BEDTools suite (Quinlan and Hall, 2010). The intersect bed function from BEDTools was then used to identify all human genes located within these expanded deafness loci. Galaxy web platform (server: usegalaxy.org; The Galaxy Community, 2024) was employed for this analysis. Human orthologs of Six1 targets identified in chick were extracted using the BioMart web-based data-mining tool. Finally, Fisher's exact one-tailed statistical test (α=0.1) was performed to determine whether the enrichment of putative Six1 targets in deafness loci is statistically significant.

## Supplementary Material



10.1242/develop.204533_sup1 Supplementary information

Table S1. Identification of putative Six1 targets in chick.

Table S2. Quantification of functional experiments and enhancer activity in chick.

Table S3. Gene expression in human CS13-14 otic vesciles and association of putative Six1 targets to know deafness loci.

Table S4. OMIM deafness genes and conservation of enhancers the in human genome.
