# Supplementary material for: Targets of the transcription factor Six1 identify previously unreported candidate deafness genes
Source: Development. 2025 Apr 14;152(7):dev204533. doi: 10.1242/dev.204533 (PMC12045605; doi:10.1242/dev.204533)
Supplement: Supplementary information [file develop-152-204533-s1.pdf]

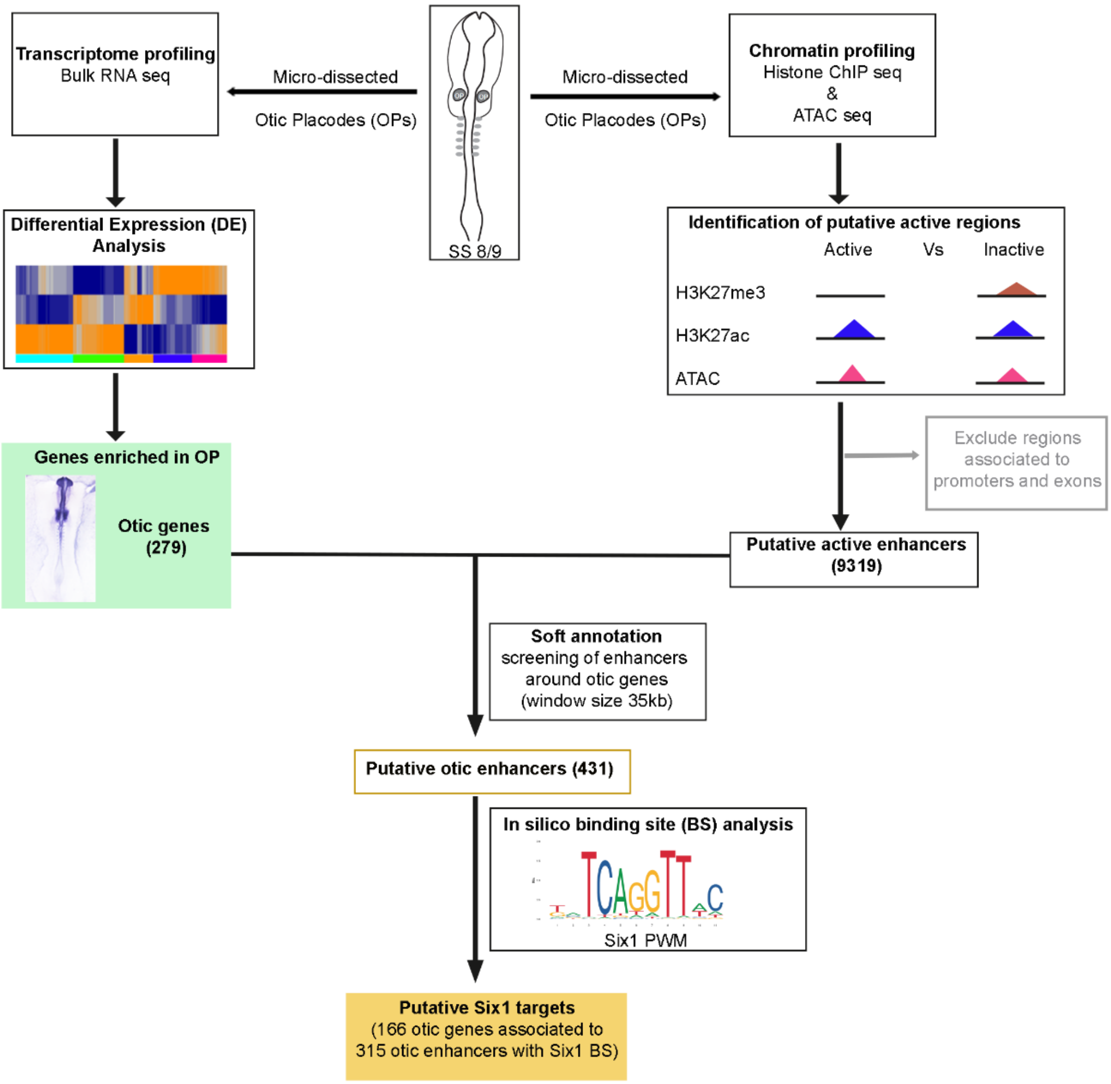

Fig. S1. Workflow to identify putative Six1 targets in chick otic progenitors. Otic placode enriched genes were taken from RNAseq from ss8-9 otic progenitors compared to ss3 embryo (Chen et al., 2017) (fold change >2, RPKM >10); a combination of ChIPseq for histone marks and ATACseq was used to identify active enhancers (Buzzi et al., 2022). Enhancers were associated to genes enriched in the otic placode, and then screened for Six1 binding motifs. This resulted in a total of 166 transcripts associated with 315 enhancers containing one or more Six1 motifs.

Development • Supplementary information

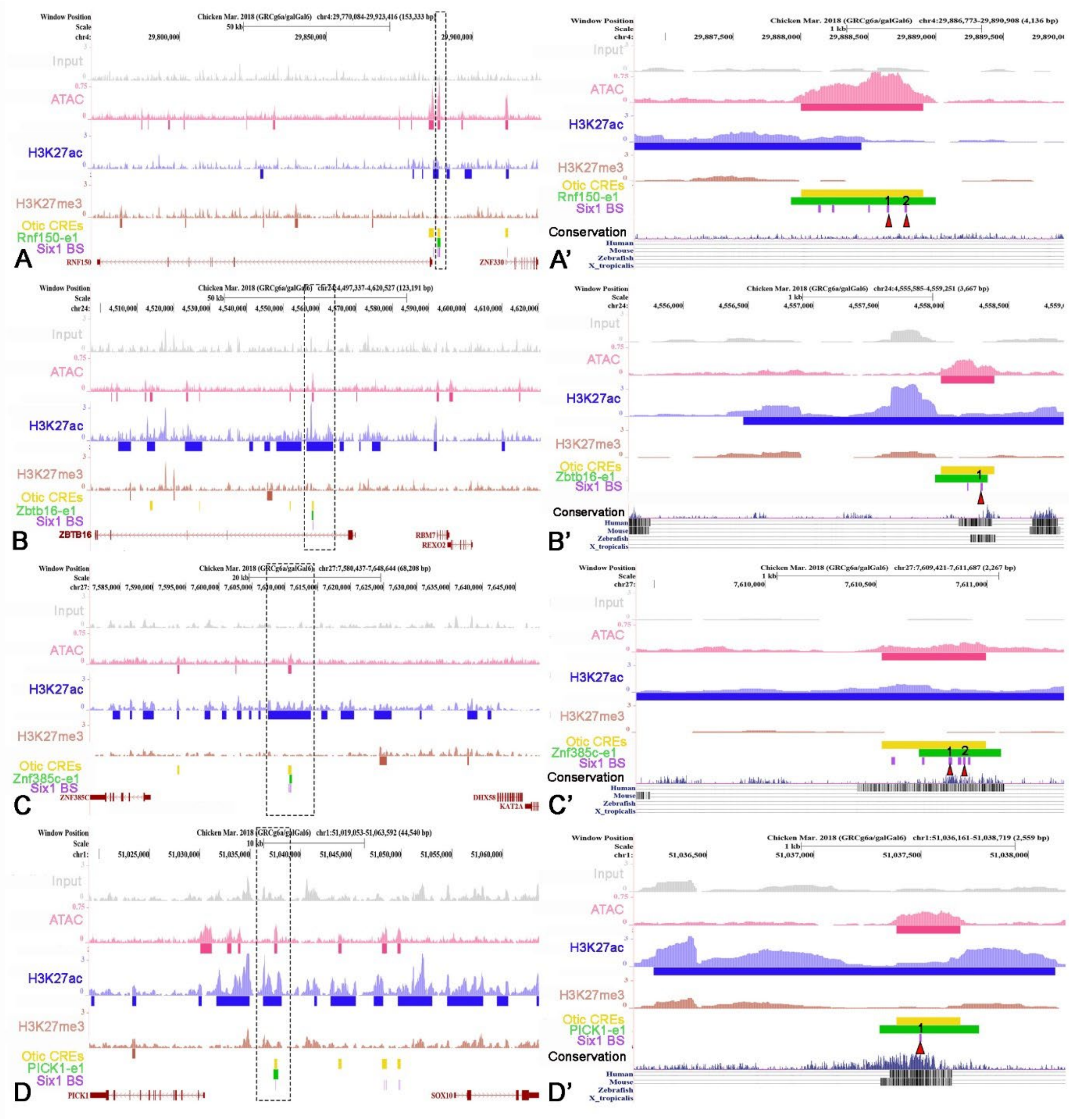

Fig. S2. Chromatin landscape of active otic placode enhancers with Six1 binding sites. A-D. show browser plots of ATACseq (pink) and ChIPseq for H3K27ac (blue) and H3K27me3 (brown) surrounding RNF150 (A), Zbtb16 (B), Znf385c (C) and Pick1 (D). A'-D' show zoomed in views of the genomic track of the boxed areas in A-D. Solid bars in pink, blue and brown underneath the tracks indicate called peaks, yellow bars predicted otic CREs, green bars regions cloned into reporter vectors and purple bars Six1 motifs. Red triangles in A'-D' indicate high fidelity Six1 binding sites which were deleted in reporter constructs. A'-D' also show sequence conservation between chick human, mouse, zebrafish and Xenopus tropicalis. The data were uploaded in the UCSC genome browser and track session link is provided in supplementary file 1

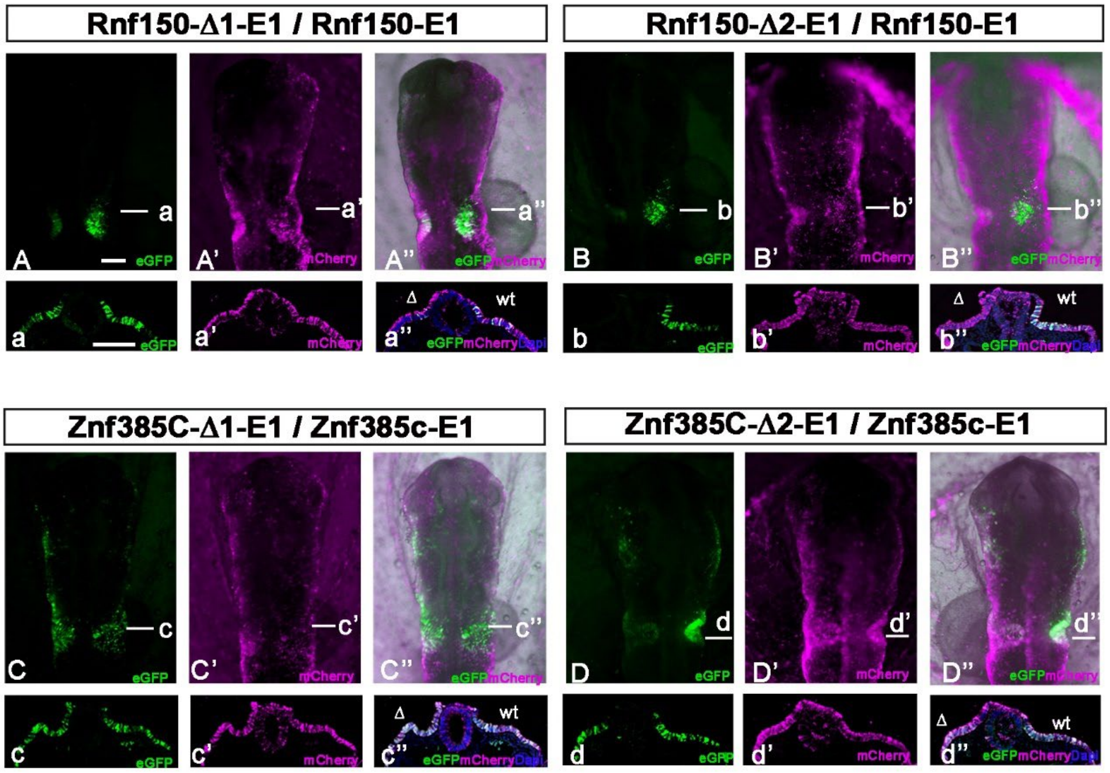

Fig. S3. The Six1 motif is required for normal enhancer activity in the otic placode. The enhancers associated to RNF150 and to ZNF385c each harbour two Six1 motifs. **A-A'', B-B''**: Deletion of each in the RNF150 associated enhancer leads to reduction of enhancer activity (left side) compared to wildtype enhancer activity (right side). A, B: eGFP in green driven by enhancer constructs; A', B': mCherry driven ubiquitously; A'', B'' overlay of both channels and bright field image. a-a'', b-b'' show sections of the embryo in A-A'' and B-B'' at the level of the white line. **C-C'', D-D''**: Deletion of Six1 motif 1 in the ZNF385c associated enhancer does not change enhancer activity, while deletion of motif 2 leads to its reduction (left side) compared to wildtype enhancer activity (right side). C, D: eGFP in green driven by enhancer constructs; C', D': mCherry driven ubiquitously; C'', D'' overlay of both channels and bright field image. d-d'', d-d'' show sections of the embryo in C-C'' and D-D'' at the level of the white line.

Development • Supplementary information

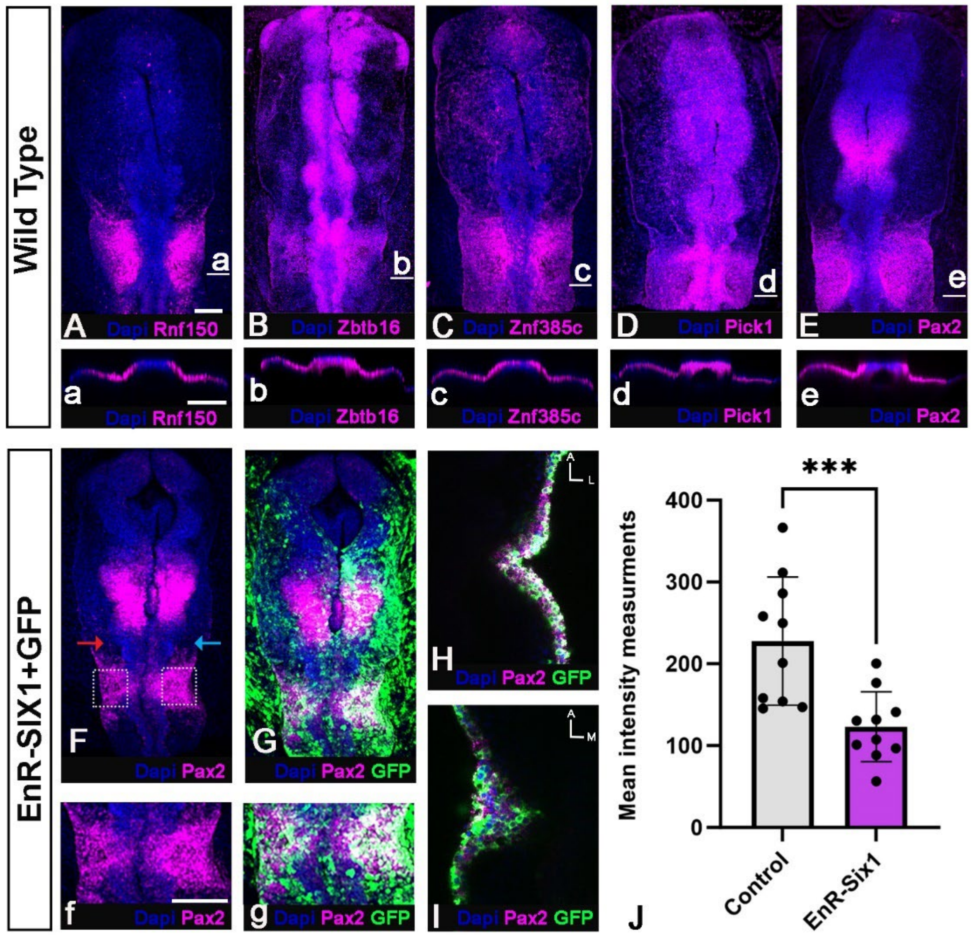

Fig. S4. Six1 regulates expression of known and novel targets in chick and Xenopus. A-E, a-e: Expression of *RNF150*, *ZBTB16*, *ZNF385c*, *Pick1* and *Pax2* in chick HH11/12 embryos assessed by in situ HCR. a-e show sections at the level of the white line in A-E. **F-J:** Six1 activity is required for the expression of *Pax2*. EnR-Six1 was coelectroporated with eGFP encoding vectors on the left side of the embryo (red arrow) and eGFP alone on the right side (blue arrow) and processed for in situ HCR for *Pax2* (F: *Pax2* expression in magenta, DAPI to visualise nuclei in blue; G: overlay *Pax2* (magenta), eGFP (green) and DAPI (blue)). **f** and **g,** zoomed in version of the otic region. **H, I:** representative image of a single confocal stack of the otic region of the embryo shown in F and G. H: control side; I: EnR-Six1. J. Quantification of *Pax2* expression in controls and EnR-Six1 electroporated embryos. Scalebars in A, a, and f are 100mm.

Development • Supplementary information

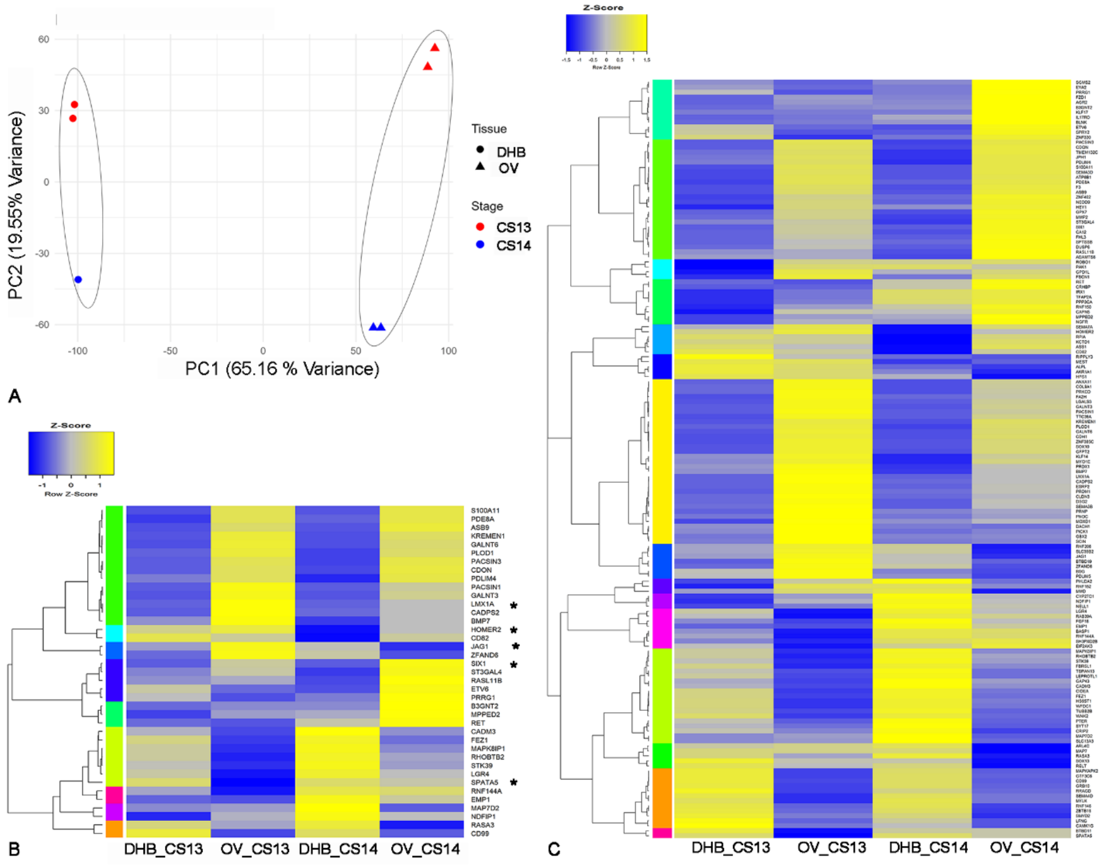

Fig. S5. RNAseq data analysis from human otic vesicles and dorsal hindbrain. A. PCA analysis of otic vesicle (triangles) and hindbrain (squares) replicates. Variance between otic vesicle and hindbrain samples is high (PC1 65%). **B.** Heatmap showing the expression of the human putative Six1 targets that fall into deafness loci. Blue: high expression; yellow: low expression. * indicates putative Six1 targets that are known deafness genes. **C.** Heatmap showing the expression of all putative human Six1 targets.

Table S1. Identification of putative Six1 targets in chick.

Available for download at
https://journals.biologists.com/dev/article-lookup/doi/10.1242/dev.204533#supplementary-data

Table S2. Quantification of functional experiments and enhancer activity in chick.

Available for download at
https://journals.biologists.com/dev/article-lookup/doi/10.1242/dev.204533#supplementary-data

Table S3. Gene expression in human CS13-14 otic vesciles and association of putative Six1 targets to know deafness loci.

Available for download at
https://journals.biologists.com/dev/article-lookup/doi/10.1242/dev.204533#supplementary-data

Table S4. OMIM deafness genes and conservation of enhancers the in human genome.

Available for download at
https://journals.biologists.com/dev/article-lookup/doi/10.1242/dev.204533#supplementary-data

Table S5. DNA plasmids for chick electroporation experiments

DNA construct	Vector	Concentration injected	Source
EGFP	pCAB-IRES-EGFP	0.5 µg/µl	(Christophorou et al., 2009)
mCherry	pCAB-IRES-mcherry	1.5 µg/µl	(Chen and Streit, 2015)
ZBTB16_E-WT	pTK-EGFP-Tag1	2 µg/µl	Cloned for this study
ZBTB16_E-Δ	pTK-EGFP-Tag1	2 µg/µl	Cloned for this study
ZNF385C_E-WT	pTK-EGFP-Tag1	2 µg/µl	Cloned for this study
ZNF385C_E-Δ1	pTK-EGFP-Tag1	2 µg/µl	Cloned for this study
ZNF385C_E-Δ2	pTK-EGFP-Tag1	2 µg/µl	Cloned for this study
ZNF385C_E-Δ1+2	pTK-EGFP-Tag1	2 µg/µl	Cloned for this study
RNF150_E-WT	pTK-EGFP-Tag8	2 µg/µl	Cloned for this study
RNF150_E-Δ1	pTK-EGFP-Tag8	2 µg/µl	Cloned for this study
RNF150_E-Δ2	pTK-EGFP-Tag8	2 µg/µl	Cloned for this study
RNF150_E-Δ1+2	pTK-EGFP-Tag8	2 µg/µl	Cloned for this study
PICK1_E-WT	pTK-EGFP-Tag7	2 µg/µl	Cloned for this study
PICK1_E-Δ	pTK-EGFP-Tag7	2 µg/µl	Cloned for this study
EnR-Six1	pCS2+	2.5 µg/µl	(Brugmann et al., 2004)

Table S6. DNA plasmids used to generate DIG labelled RNA probes for chick wholemount in situ hybridisation.

Gene name	Vector	Insert size	Restriction enzyme	RNA pol	Plasmid Source
PICK1	pBluescript IIKS+	406	XhoI	T7	Synthesized from Invitrogen
RNF150	pBluescript IIKS+	404	XhoI	T7	Synthesized from Invitrogen
SIX1	pBluescript IIsk+	500	BamHI/ SmaI	T3	(Christophorou et al., 2009)
ZBTB16	pBluescript IIKS+	594	-	T3	(Chen et al., 2017)
ZNF385C	TOPO pCR2.1	389	-	T7	(Chen et al., 2017)

Development • Supplementary information

Table S7. *Xenopus* DNA plasmids used to generate DIG labelled RNA probes for WISH or mRNA for embryo microinjection. Plasmids encoding Pick1, Rnf150, Zbtb16 and Znf385c were purchased from the *Xenopus* Gene Collection (Klein et al. 2002). Subsequently, these plasmids were subcloned into Gateway vectors as part of the ORFeome project (Grant et al., 2015), and are now available from Horizon Discovery Ltd. in their Xenopus Collection (https://horizondiscovery.com/en/non-mammalian-research-tools/products/xenopus-collection).

**Gene name** (assay)	**Vector**	**Insert size** (bp)	**Restriction enzyme**	**RNA pol**	**Plasmid Source and Clone ID** (species)
**Pick1** (WISH)	pCMV-SPORT6	1218	EcoRV	T7	Dharmacon #7764462 (*X. laevis*)
**Rnf150** (WISH)	pCMV-SPORT6	1500	KpnI	T7	Dharmacon #7641707 (*X. tropicalis*)
**Six1** (WISH)	pBluescript II SK+	1400	NotI	T7	(Pandur and Moody, 2000) (*X. laevis*)
**EnR-Six1** (Microinjection)	pCS2+	1398	Asp718	SP6	(Brugmann et al., 2004) (*X. laevis*)
**Zbtb16** (WISH)	pCMV-SPORT6	600	StuI	T7	Source BioScience #IMAGp998F2016230Q (*X. tropicalis*)
**Znf385c** (WISH)	pCMV-SPORT6	1500	StuI	T7	Dharmacon #7687943 (*X. tropicalis*)

Brugmann, S.A., Pandur, P.D., Kenyon, K.L., Pignoni, F., Moody, S.A., 2004. Six1 promotes a placodal fate within the lateral neurogenic ectoderm by functioning as both a transcriptional activator and repressor. *Development* **131**, 5871-5881.

Buzzi, A.L., Chen, J., Thiery, A., Delile, J., Streit, A., 2022. Sox8 is sufficient to reprogram ectoderm into ear vesicles and associated neurons. *Proc Natl Acad Sci U S A* **119**, e2118938119.

Chen, J., Streit, A., 2015. A medium-scale assay for enhancer validation in amniotes. *Developmental dynamics : an official publication of the American Association of Anatomists* **244**, 1291-1299.

Chen, J., Tambalo, M., Barembaum, M., Ranganathan, R., Simoes-Costa, M., Bronner, M.E., Streit, A., 2017. A systems-level approach reveals new gene regulatory modules in the developing ear. *Development* **144**, 1531-1543.

Christophorou, N.A., Bailey, A.P., Hanson, S., Streit, A., 2009. Activation of Six1 target genes is required for sensory placode formation. *Dev Biol* **336**, 327-336.

Pandur, P.D., Moody, S.A., 2000. Xenopus Six1 gene is expressed in neurogenic cranial placodes and maintained in the differentiating lateral lines. *Mech Dev* **96**, 253-257.

Development • Supplementary information
