# Supplementary material for: Targets of the transcription factor Six1 identify previously unreported candidate deafness genes
Source: Development. 2025 Apr 14;152(7):dev204533. doi: 10.1242/dev.204533 (PMC12045605; doi:10.1242/dev.204533)
Supplement: Supplementary file 1 [file develop-152-204533_review_history.pdf]

Direct targets of the transcription factor Six1 identify novel candidate deafness genes

Andrea Streit, Ramya Ranganathan, Fereshteh Sari, Scarlet Xiaoyan Wang, Alexandre Thiery, Ailin Leticia Buzzi, Rosalinda Guerra and Sally Moody

DOI: 10.1242/dev.204533

Editor: Benoit G. Bruneau

Review timeline

Original submission: 18 November 2024
Editorial decision: 13 December 2024
First revision received: 11 February 2025
Accepted: 12 February 2025

Original submission

First decision letter

MS ID#: dev.204533

MS TITLE: Direct targets of the transcription factor Six1 identify novel candidate deafness genes

AUTHORS: Andrea Streit, Ramya Ranganathan, Fereshteh Sari, Scarlet Xiaoyan Wang, Alexandre Thiery, Ailin Leticia Buzzi, Rosalinda Guerra and Sally Moody

I have now received all the referees' reports on the above manuscript, and have reached a decision. The referees' comments are appended below, or you can access them online: please go to:

As you will see, the referees express considerable interest in your work, but have some significant criticisms and recommend a substantial revision of your manuscript before we can consider publication. If you are able to revise the manuscript along the lines suggested, which may involve further experiments, I will be happy receive a revised version of the manuscript. Your revised paper will be re-reviewed by one or more of the original referees, and acceptance of your manuscript will depend on your addressing satisfactorily the reviewers' major concerns. Please also note that Development will normally permit only one round of major revision. If it would be helpful, you are welcome to contact us to discuss your revision in greater detail. Please send us a point-by-point response indicating your plans for addressing the referees’ comments, and we will look over this and provide further guidance.

Please attend to all of the reviewers' comments and ensure that you clearly highlight all changes made in the revised manuscript. Please avoid using 'Tracked changes' in Word files as these are lost in PDF conversion. I should be grateful if you would also provide a point-by-point response detailing how you have dealt with the points raised by the reviewers in the 'Response to Reviewers' box. If you do not agree with any of their criticisms or suggestions please explain clearly why this is so.

SUMMARY OF THE ADVANCE MADE IN THIS PAPER AND ITS POTENTIAL SIGNIFICANCE TO THE FIELD

© 2025. Published by The Company of Biologists under the terms of the Creative Commons Attribution License (https://creativecommons.org/licenses/by/4.0/).

Six1 plays a crucial role in cranial placode development, and mutations in the SIX1 gene are consistently associated with Branchio-Oto-Renal (BOR) syndrome in human patients. In their manuscript, Ranganathan et al. analyzed the targets of Six1 during otic development. They identify a set of putative Six1 target genes and further validate the regulation of four of these targets in both Chick and Xenopus models. Additionally, the authors provide RNA-seq data from the developing human ear, suggesting that some of these Six1 targets could potentially represent novel deafness genes. Overall, the experiments are well-designed and executed.

SUGGESTIONS TO AUTHORS

1. Figure 2 and Supplementary Figure S3: The enhancer activity presented in these figures should be quantified to provide a more rigorous and quantitative assessment of their findings.
2. Figure 3: The authors dedicate an entire figure to demonstrating how Six1 regulates the four target genes in the Xenopus ear. However, the results appear complex, as both overexpression and downregulation of Six1 lead to a reduction in the expression of these targets. This unexpected outcome warrants further discussion in the manuscript's Discussion section to explore potential mechanisms underlying these observations.
3. Scale bars in figures: None of the figures in the manuscript include scale bars. The authors should ensure scale bars are added to all relevant figures for clarity and reproducibility.

SUMMARY OF THE ADVANCE MADE IN THIS PAPER AND ITS POTENTIAL SIGNIFICANCE TO THE FIELD

This quite interesting manuscript aims to connect investigation of a gene regulatory network guiding ear development to identification of new candidate deafness genes. The starting point is the transcription factor Six1, mutations of which account for a significant proportion of autosomal dominant Branchio-otic and Branchio-oto-renal syndrome cases. The authors use a combination of Xenopus and chick experiments to identify and test Six1 target genes and associated cis-regulatory elements, and end with an exploration of human candidate Six1 targets that are associated with deafness loci. For the most part, the experimental design seems sufficiently rigorous and the data are sufficiently strong to support the main conclusions. There is a good bit of new information in this manuscript, and the human data represent an important advancement building from the experimental models work. There are some rough spots in the manuscript that need to be cleaned up, but I think these can be addressed through clarifications to the writing and shouldn't require additional experiments.

SUGGESTIONS TO AUTHORS

1. The authors use "Xenopus" throughout the text to refer to frog experiments, but they use both laevis and tropicalis without identifying which they use for each experiment. The description of the tropicalis work is particularly problematic. Specifically, in Figure 3 are these all tropicalis, or are they all laevis except for those in the column labeled "null"? Are "controls" wild-type siblings of "nulls"? How are the mutant embryos produced? Are they from intercrosses of heterozygous carriers? If so, how do they know that these are the homozygous mutants? Are they genotyped? Also, despite the fact that they have a prior manuscript with "null" in the title, this is an improper way to describe this allele. I found nothing in the Coppenrath paper that shows that this allele is "null", a term that has a specific meaning. Instead, this is better described as a loss-of-function mutation. It might be a total loss-of-function and, therefore, null, but it is misleading to refer to it as such without the proper experiments and supporting evidence.

2. There is no citation for the anti-Six1 antibody used for ChIP, only a vendor source. Has it been validated in any way? This should be noted.

© 2025. Published by The Company of Biologists under the terms of the Creative Commons Attribution License (https://creativecommons.org/licenses/by/4.0/).

3. The authors pick out 4 genes that are co-expressed with Six1 in the otic vesicles, consistent with the possibility that Six1 promotes their expression. Most of their cis-regulatory element work support that possibility but there is one exception. Deletion of the Six1 motif in Zbtb16-E1-eGFP increases reporter gene expression, suggesting that this element is a silencer rather than an enhancer. From this the authors state (line 111) "proposing that Six1 acts as a repressor of Zbtb16". However, the other data in this paper indicate that Six1 promotes Zbtb16 expression. The authors seem to ignore this incongruity, but they really need to address is somehow.
4. There is no quantification provided for the chick experiments shown in Figure 2C-F. The data might be included in Supplementary Table 2 but I can't be sure because the nomenclature doesn't match. In any case, there should be some sort of information in the figure/figure legend to show frequency of phenotypes and number of embyros/experiments.
5. The data of Figure 3 are baffling to me, because they show that loss-of-function and gain-of-function manipulations have similar effects. The authors address this (lines 145-148) by saying, in effect, "it's complicated" but this is very unsatisfactory. I have a concern here that the experimental tools are not doing what the authors think they are doing. Indeed, there essentially are no validations provided in this manuscript, although several reagents have been published. Ultimately, I just don't think that the Six1 mRNA, EnR-Six1, and VP16-Six1 data provide anything very useful.
6. On lines 163-164 the authors state, "…in ear progenitors, and that it does so by interacting directly with their respective enhancers." This is a bit of an overstatement because the authors are missing a key piece of data to support that conclusion. They would need to delete putative cis-regulatory elements in vivo to support this conclusion.

Reviewer 3:

The manuscript by Streit et al. aims at identifying novel direct transcriptional targets of the transcription factor Six1 in chick, Xenopus and human in the developing inner ear as these are good candidates to be responsible to deafness related syndromes.
The authors identify genes specifically expressed in the chick inner ear and including putative binding sites for the transcription factor Six1 in their cis regulatory region. They further characterize 4 of those genes and their cis-regulatory regions. These results together with changes in expression levels of those 4 genes following manipulations of Six1 activity in chick and Xenopus provide evidence that they are likely direct downstream targets of Six1 in chick and that they are regulated by Six1 in Xenopus.
A second part relates to the identification of human genes expressed in the otic vesicle that are orthologs of the Six1 targets identified in chick. 36 of these genes are located nearby loci previously associated to deafness where the causative gene has not been determined yet and it is proposed that they are good candidates to be affected in deafness.
I consider the identification of good candidate genes related to deafness as an important advance towards understanding the disease. However, although the possible involvement of the candidates in several relevant pathways in development is highlighted and discussed, this is not assessed in the article and the significant and novel contribution to the understanding of developmental mechanisms is not clear.

SUGGESTIONS TO AUTHORS
The evidence provided in the manuscript is thorough, good quality and sufficient to justify the general conclusion of the identification of candidate genes but this conclusion needs to be revised throughout the text so that the difference between direct targets (those where binding and regulation of transcription as well as effect of mutation in the binding site has been shown) vs candidate targets is made clearer.
There are issues that I consider should be addressed:
(1) As indicated above, my major concern is the confusion throughout the text of what is considered as demonstrated and what is suggested. For example, with respect to the consideration of Six1 targets as proven or putative/candidate, as for some of the targets have been examined in more detail than for others.

© 2025. Published by The Company of Biologists under the terms of the Creative Commons Attribution License (https://creativecommons.org/licenses/by/4.0/).

For example, in the abstract: "Using published transcriptomic and epigenomic data from chick ear progenitors, we first identify putative Six1 targets. Next, we provide evidence that Six1 directly regulates these targets…" seems to imply that all those targets initially identified as candidates have been further assessed, when this is not the case.
The text must be revised to avoid considering suggestions as demonstrations. For example, in lines 58, 194, 265, I suggest to:
Line 58: change "Six1 targets" for "Six1 putative targets"
Line 194: change "…the Six1 targets identified in chick are likely to mediate its function in human ear progenitors" change "are likely" to "are good candidates"
Line 265: change "…as direct targets" to "…as putative direct target."
(2) With respect to statistical reporting:
Line 100: If the mean of the measure of the binding of Six1 to ZNF385C is above the control but the significance test performed gives no significance (according to the criterium indicated by the authors), it is not possible to conclude that the binding is enhanced but not significant. The appropriate conclusion is that "there is no evidence of binding".
Line 104: change "best p-value" with "lowest p-value".
(3) Relating to Xenopues data:
It needs to be made clear that although they indicate a relation of Six1 and the candidate target genes, the direct regulation is not demonstrated and seems to be more complex. This should be clarified in Line 162 where it is implied that the relation is direct.

Figure 3: The quantification in panels U-X is not clear, what is being quantified? The size/lack or otic vesicle only? Is there a quantification of the gene expression level? Are the transcripts downregulated in cases where the otic vesicle is normal in size? Is this only a control that the procedure with respect to size of the otic vesicle is reproducible across experiments? This needs to be clarified in the figure legend.

As the constructs used for interfering with Six1 have been described before, the reference should be included in the Results.

(4) Relating to chick data
In Figure 3, the sections showing gene expression following electroporation of control or Six1-EnR construct, appear morphologically different in the 2 sides (for example panel R vs panel S), is this so? Or are they framed differently?
Indicate the microscope, objective numerical aperture and Z-step size of the images used for quantification of HCR3 signal.

(5) With respect to human data:
Figure 4: The part about human data could be moved to a different figure for clarity.
Line 198: With respect to the human loci that have been previously related to deafness and where a responsible gene has been identified (185 loci), reference to the article(s) where this has been shown should be included.
Relating to the conserved CREs shown for 3 human candidate genes, does this conservation include the Six1 putative binding sites found in chick? This should be clarified.
Line 296: It is not clear why the 2 genes that are highlighted in the discussion as new candidates for BOR/BOS are not included in the results section where the 36 genes candidates for deafness are explained. Other Six1 candidates are mentioned in the results such as Kremen or LGR4. It is not clear why some are specifically mentioned in the results and others only in the discussion, can this difference be explained? Also, I could not find the gene SPAK in the SuppFile3.xlx.

First revision

Author response to reviewers' comments

© 2025. Published by The Company of Biologists under the terms of the Creative Commons Attribution License (https://creativecommons.org/licenses/by/4.0/).

We thank the reviewers for their constructive comments and have addressed their points below

Reviewer 1: SUMMARY OF THE ADVANCE MADE IN THIS PAPER AND ITS POTENTIAL SIGNIFICANCE TO THE FIELD

Six1 plays a crucial role in cranial placode development, and mutations in the SIX1 gene are consistently associated with Branchio-Oto-Renal (BOR) syndrome in human patients. In their manuscript, Ranganathan et al. analyzed the targets of Six1 during otic development. They identify a set of putative Six1 target genes and further validate the regulation of four of these targets in both Chick and Xenopus models. Additionally, the authors provide RNA-seq data from the developing human ear, suggesting that some of these Six1 targets could potentially represent novel deafness genes. Overall, the experiments are well-designed and executed.

SUGGESTIONS TO AUTHORS
1. Figure 2 and Supplementary Figure S3: The enhancer activity presented in these figures should be quantified to provide a more rigorous and quantitative assessment of their findings.
We have quantified the enhancer activity as requested, added graphs into Fig. 2. and quantification data into supplementary file 2, as well as embryo numbers in the text and figure legend.

2. Figure 3: The authors dedicate an entire figure to demonstrating how Six1 regulates the four target genes in the Xenopus ear. However, the results appear complex, as both overexpression and downregulation of Six1 lead to a reduction in the expression of these targets. This unexpected outcome warrants further discussion in the manuscript's Discussion section to explore potential mechanisms underlying these observations.
We agree with the reviewer that the results as originally presented are complex. The main point we wanted to address is whether Six1 actually regulates our candidate genes. We have therefor revised Figure 3 extensively to focus on whether Six1 is required for the expression of the four genes of interest, and to address comments from the other reviewers (R2, point 5; R3, point 3). We now present only the results from two different methods for reducing Six1 levels in the embryo: injections of Six1 MOs in *X. laevis*, and Six1 loss-of-function Crispants in *X. tropicalis*. We also present injections of mRNA encoding the repressive *EnR-Six1* in *X. laevis*, which represses all target genes constitutively. Together these data provide convincing support that Six1 is required for the expression of the four genes, leading us to suggest that in both *X. leavis* and *X. tropicalis* Six1 is an upstream regulator of each. However, these data do not address whether the regulation is direct or indirect - which is addressed with the experiments in chick (Figures 2, 4 and S3) - which we now clearly state in the text.

3. Scale bars in figures: None of the figures in the manuscript include scale bars. The authors should ensure scale bars are added to all relevant figures for clarity and reproducibility.
We apologize and have corrected this oversight.

Reviewer 2: SUMMARY OF THE ADVANCE MADE IN THIS PAPER AND ITS POTENTIAL SIGNIFICANCE TO THE FIELD
This quite interesting manuscript aims to connect investigation of a gene regulatory network guiding ear development to identification of new candidate deafness genes. The starting point is the transcription factor Six1, mutations of which account for a significant proportion of autosomal dominant Branchio-otic and Branchio-oto-renal syndrome cases. The authors use a combination of Xenopus and chick experiments to identify and test Six1 target genes and associated cis-regulatory elements, and end with an exploration of human candidate Six1 targets that are associated with deafness loci. For the most part, the experimental design seems sufficiently rigorous and the data are sufficiently strong to support the main conclusions. There is a good bit of new information in this manuscript, and the human data represent an important advancement building from the experimental models work. There are some rough spots in the manuscript that need to be cleaned up, but I think these can be addressed through clarifications to the writing and shouldn't require additional experiments.

© 2025. Published by The Company of Biologists under the terms of the Creative Commons Attribution License (https://creativecommons.org/licenses/by/4.0/).

SUGGESTIONS TO AUTHORS

1. The authors use "Xenopus" throughout the text to refer to frog experiments, but they use both laevis and tropicalis without identifying which they use for each experiment. The description of the tropicalis work is particularly problematic. Specifically, in Figure 3 are these all tropicalis, or are they all laevis except for those in the column labeled "null"? Are "controls" wild-type siblings of "nulls"? How are the mutant embryos produced? Are they from intercrosses of heterozygous carriers? If so, how do they know that these are the homozygous mutants? Are they genotyped? Also, despite the fact that they have a prior manuscript with "null" in the title, this is an improper way to describe this allele. I found nothing in the Coppenrath paper that shows that this allele is "null", a term that has a specific meaning. Instead, this is better described as a loss-of-function mutation. It might be a total loss-of-function and, therefore, null, but it is misleading to refer to it as such without the proper experiments and supporting evidence.
a) We thank the reviewer for catching this lapse. We reformatted Figure 3 in response to the comments of all reviewers. In the new Figure 3A-D are the uninjected sides of Six1 morphants, and A'-D' are the injected sides of the same larvae; these are wildtype *Xenopus laevis*. The embryos injected with EnR-Six1 mRNA (3E-H) are also *Xenopus laevis*. We now specify this in the figure legend and text (page 8, lines 145-164). Those previously labeled "nulls" are *Xenopus tropicalis* Crispants. This is now specified in the figure legend and text (page 8, lines 159-161). Also, the term null has been replaced with "Six1 loss-of-function mutants" created by CRISPR-Cas9 or as "Crispants".
b) The reviewer is correct in that the Six1 Crispants are not technically nulls but loss-of-function mutants; the protocol results in a stop codon upstream of the DNA-binding domain. We have now corrected the description of these larvae in the legend for Figure 3 and in the text (page 8, lines 159-161).
2. There is no citation for the anti-Six1 antibody used for ChIP, only a vendor source. Has it been validated in any way? This should be noted.

The vendor source was listed in the methods section; we have now added the appropriate citation Li et al., 2020, who have used this antibody for ChIP-seq in the mouse ear.

3. The authors pick out 4 genes that are co-expressed with Six1 in the otic vesicles, consistent with the possibility that Six1 promotes their expression. Most of their cis-regulatory element work support that possibility but there is one exception. Deletion of the Six1 motif in Zbtb16-E1-eGFP increases reporter gene expression, suggesting that this element is a silencer rather than an enhancer. From this the authors state (line 111) "proposing that Six1 acts as a repressor of Zbtb16". However, the other data in this paper indicate that Six1 promotes Zbtb16 expression. The authors seem to ignore this incongruity, but they really need to address is somehow.

The reviewer is correct that these results appear to be contradictory, and that we have not provided any explanation. The regulatory relationships are likely to be complex and our experiments are not designed to disentangle the entire transcriptional cascade. We would like to stress that the main point of our study is to identify Six1 targets, irrespective of whether they are activated or repressed. Depending on co-factor availability Six1 acts as a transcriptional activator or repressor, and the experiments presented here cannot distinguish this. This would involve considerable additional work including biochemistry and co-factor identification from embryonic material. We have now clarified this throughout the text.

Related to Zbtb16 there are different scenarios that might explain our results. However, to disentangle the precise regulatory relationship between Six1 and Zbtb16 requires many complex experiments beyond the scope of this manuscript. We have now expanded the discussion and discuss different potential explanations for the apparent incongruity (3rd paragraph of the discussion).

4. There is no quantification provided for the chick experiments shown in Figure 2C-F. The data might be included in Supplementary Table 2 but I can't be sure because the nomenclature doesn't

© 2025. Published by The Company of Biologists under the terms of the Creative Commons Attribution License (https://creativecommons.org/licenses/by/4.0/).

match. In any case, there should be some sort of information in the figure/figure legend to show frequency of phenotypes and number of embryos/experiments.
We apologize for this oversight and for the mismatch of the nomenclature. We have now edited the figure and supplementary file with the correct nomenclature, added embryo numbers in the text and legend for Figure 2. We also provide quantification of the activity of the wildtype and deletion construct activities (see reviewer 1 point 1).

5. The data of Figure 3 are baffling to me, because they show that loss-of-function and gain-of-function manipulations have similar effects. The authors address this (lines 145-148) by saying, in effect, "it's complicated" but this is very unsatisfactory. I have a concern here that the experimental tools are not doing what the authors think they are doing. Indeed, there essentially are no validations provided in this manuscript, although several reagents have been published. Ultimately, I just don't think that the Six1 mRNA, EnR-Six1, and VP16-Six1 data provide anything very useful.
We agree with the reviewer that some of the mRNA data do not add clarity to the question of whether Six1 acts upstream of the putative target genes of interest. Therefore, we have reorganized Figure 3 to focus on three loss-of function manipulations, as described in the response to R1, point 2, all of which demonstrate that Six1 is required for their expression.

For nearly 30 years, the *Xenopus* EnR construct has been extensively characterized and published in the literature (Conlon et al., 1996; Fan and Sokol, 1997) and has been a standard method for creating repressive versions of transcription factors. For example, see: Sive et al., 2000, Early Development of *Xenopus laevis*: a Laboratory Manual, Cold Spring Harbor Press; LaBonne and Bronner-Fraser, Dev Biol 2000; Pohl and Knochel, Mech Dev 2001; Goto et al., PNAS 2010; Huber et al., Current Biol 2001; Kumar et al., Cells 2023; Pownall et al., Current Biol. 1998; and many more. However, since transcriptional activation by Six1 has been reported to require binding to certain cofactor proteins (e.g., Pignoni et al., Cell 1997), we agree with the reviewer that the VP16-Six1 construct may not be acting as expected. Therefore, we have removed these data from the manuscript.

6. On lines 163-164 the authors state, "…in ear progenitors, and that it does so by interacting directly with their respective enhancers." This is a bit of an overstatement because the authors are missing a key piece of data to support that conclusion. They would need to delete putative cis-regulatory elements in vivo to support this conclusion.
We agree with the reviewer and have deleted this statement.

Reviewer 3:
The manuscript by Streit et al. aims at identifying novel direct transcriptional targets of the transcription factor Six1 in chick, Xenopus and human in the developing inner ear as these are good candidates to be responsible to deafness related syndromes.
The authors identify genes specifically expressed in the chick inner ear and including putative binding sites for the transcription factor Six1 in their cis regulatory region. They further characterize 4 of those genes and their cis-regulatory regions. These results together with changes in expression levels of those 4 genes following manipulations of Six1 activity in chick and Xenopus provide evidence that they are likely direct downstream targets of Six1 in chick and that they are regulated by Six1 in Xenopus.
A second part relates to the identification of human genes expressed in the otic vesicle that are orthologs of the Six1 targets identified in chick. 36 of these genes are located nearby loci previously associated to deafness where the causative gene has not been determined yet and it is proposed that they are good candidates to be affected in deafness.
I consider the identification of good candidate genes related to deafness as an important advance towards understanding the disease. However, although the possible involvement of the candidates in several relevant pathways in development is highlighted and discussed, this is not assessed in the article and the significant and novel contribution to the understanding of developmental mechanisms is not clear.

© 2025. Published by The Company of Biologists under the terms of the Creative Commons Attribution License (https://creativecommons.org/licenses/by/4.0/).

SUGGESTIONS TO AUTHORS
The evidence provided in the manuscript is thorough, good quality and sufficient to justify the general conclusion of the identification of candidate genes but this conclusion needs to be revised throughout the text so that the difference between direct targets (those where binding and regulation of transcription as well as effect of mutation in the binding site has been shown) vs candidate targets is made clearer.
Thanks for pointing this out; we have made it clear in the text and have reworded as suggested below.

There are issues that I consider should be addressed:
(1) As indicated above, my major concern is the confusion throughout the text of what is considered as demonstrated and what is suggested. For example, with respect to the consideration of Six1 targets as proven or putative/candidate, as for some of the targets have been examined in more detail than for others.
For example, in the abstract: "Using published transcriptomic and epigenomic data from chick ear progenitors, we first identify putative Six1 targets. Next, we provide evidence that Six1 directly regulates these targets…" seems to imply that all those targets initially identified as candidates have been further assessed, when this is not the case.
The text must be revised to avoid considering suggestions as demonstrations. For example, in lines 58, 194, 265, I suggest to:
Line 58: change "Six1 targets" for "Six1 putative targets"
Line 194: change "…the Six1 targets identified in chick are likely to mediate its function in human ear progenitors" change "are likely" to "are good candidates"
Line 265: change "…as direct targets" to "…as putative direct target."
We have carefully scanned the text and added ‘putative’ or ‘candidate’ targets, or similar when appropriate.

(2) With respect to statistical reporting:
Line 100: If the mean of the measure of the binding of Six1 to ZNF385C is above the control but the significance test performed gives no significance (according to the criterium indicated by the authors), it is not possible to conclude that the binding is enhanced but not significant. The appropriate conclusion is that "there is no evidence of binding".
We agree with the reviewer and have removed this statement.
Line 104: change "best p-value" with "lowest p-value".
Done.

(3) Relating to Xenopus data:
It needs to be made clear that although they indicate a relation of Six1 and the candidate target genes, the direct regulation is not demonstrated and seems to be more complex. This should be clarified in Line 162 where it is implied that the relation is direct.
Agreed. We have revised the statement to read that the reduced expression of Rnf150, Zbtb16, ZNF385c and Pick1 after three different methods of reducing Six1 indicate that Six1 is required for their expression, and suggest it is an upstream regulator, but they do not address whether the regulation is direct or indirect.

Figure 3: The quantification in panels U-X is not clear, what is being quantified? The size/lack or otic vesicle only? Is there a quantification of the gene expression level? Are the transcripts downregulated in cases where the otic vesicle is normal in size? Is this only a control that the procedure with respect to size of the otic vesicle is reproducible across experiments? This needs to be clarified in the figure legend.
We agree that there was insufficient description provided to interpret the graphs in the previous version of Figure 3. Instead of graphs, we now report the percentage of cases showing reduced gene expression in the otic vesicle, regardless of the size of the otic vesicle, in the text (page 8, lines 152-154). The percentage of cases in which the otic vesicle was reduced in size is separately reported in the text for morphants (p8, line 154) and EnR-Six1 (page 8, lines 155-158).The figure and its legend have been totally revised (see also response to R1, point 2 and R2, point 5).

© 2025. Published by The Company of Biologists under the terms of the Creative Commons Attribution License (https://creativecommons.org/licenses/by/4.0/).

As the constructs used for interfering with Six1 have been described before, the reference should be included in the Results.
The reference for both Six1 MOs and EnR-Six1 (Brugmann et al., 2004) is now included in the Results. Additional references have been added to the Methods section.

(4) Relating to chick data
In Figure 3, the sections showing gene expression following electroporation of control or Six1-EnR construct, appear morphologically different in the 2 sides (for example panel R vs panel S), is this so? Or are they framed differently?
Indicate the microscope, objective numerical aperture and Z-step size of the images used for quantification of HCR3 signal.
We think the reviewer refers to Figure 4. Indeed it is correct that the placodes change morphology after Six1-EnR expression with cells expressing the construct not forming normal placode morphology. We already reported this in a previous paper Christophorou et al., 2009. The images show z-stacks of the dotted box in whole mounts.
The images were captured using LSM850 confocal microscopy at 10x and 40x. For quantifications the images were acquired as 40x with a digital zoom 0.6x and z-stack step size is 5mm. This is now mentioned in the methods.

(5) With respect to human data:
Figure 4: The part about human data could be moved to a different figure for clarity.
We thank the reviewer for this suggestion and agree. We have now made a new Figure 5 for the human data and have also added the heatmap showing the expression of putative Six1 targets that are located in known deafness loci.

Line 198: With respect to the human loci that have been previously related to deafness and where a responsible gene has been identified (185 loci), reference to the article(s) where this has been shown should be included.
Including the references for each of these loci is not possible as there are many for each locus; these data were extracted from the OMIM database as explained in the methods. We now have added a new supplementary file (Supp. File 4) which contains all known deafness loci extracted from OMIM including those where causative genes have been identified.
Relating to the conserved CREs shown for 3 human candidate genes, does this conservation include the Six1 putative binding sites found in chick? This should be clarified.
This is a good point; we have extracted the corresponding human coordinates and run a Six1 motif analysis. We now include this information in the text (page 11, lines 217-219) and in the new supplementary file 4.

Line 296: It is not clear why the 2 genes that are highlighted in the discussion as new candidates for BOR/BOS are not included in the results section where the 36 genes candidates for deafness are explained. Other Six1 candidates are mentioned in the results such as Kremen or LGR4. It is not clear why some are specifically mentioned in the results and others only in the discussion, can this difference be explained? Also, I could not find the gene SPAK in the SuppFile3.xlx.
Thanks for pointing this out, we have now added these genes to the results section.
With relation to SPAK, the nomenclature is confusing indeed. The currently approved symbol is STK38, while SPAK is an alternative symbol. Our files and the text now uses ‘STK38, also called SPAK’ as this name is used in the papers cited.

Second decision letter

MS ID#: dev.204533R1

© 2025. Published by The Company of Biologists under the terms of the Creative Commons Attribution License (https://creativecommons.org/licenses/by/4.0/).

MS TITLE: Direct targets of the transcription factor Six1 identify novel candidate deafness genes

AUTHORS: Andrea Streit, Ramya Ranganathan, Fereshteh Sari, Scarlet Xiaoyan Wang, Alexandre Thiery, Ailin Leticia Buzzi, Rosalinda Guerra and Sally Moody

Dear Dr Streit,

I am happy to tell you that your manuscript has been accepted for publication in Development, pending our standard publication integrity checks.

© 2025. Published by The Company of Biologists under the terms of the Creative Commons Attribution License (https://creativecommons.org/licenses/by/4.0/).
